# Atlas of Musculoskeletal Stem Cells with the Soft and Hard Tissue Differentiation Architecture

**DOI:** 10.1002/advs.202000938

**Published:** 2020-10-22

**Authors:** Zi Yin, Junxin Lin, Ruojin Yan, Richun Liu, Mengfei Liu, Bo Zhou, Wenyan Zhou, Chengrui An, Yangwu Chen, Yejun Hu, Chunmei Fan, Kun Zhao, Bingbing Wu, Xiaohui Zou, Jin Zhang, Ahmed H. El‐Hashash, Xiao Chen, Hongwei Ouyang

**Affiliations:** ^1^ Dr. Li Dak Sum & Yip Yio Chin Center for Stem Cells and Regenerative Medicine, and Department of Orthopedic Surgery of Sir Run Run Shaw Hospital Zhejiang University School of Medicine Hangzhou 310058 China; ^2^ Key Laboratory of Tissue Engineering and Regenerative Medicine of Zhejiang Province Zhejiang University School of Medicine Hangzhou 310058 China; ^3^ China Orthopedic Regenerative Medicine (CORMed) Hangzhou 310058 China; ^4^ Dr. Li Dak Sum & Yip Yio Chin Center for Stem Cells and Regenerative Medicine, and Department of Orthopedic Surgery of The Second Affiliated Hospital Zhejiang University School of Medicine Hangzhou 310058 China; ^5^ Zhejiang University‐University of Edinburgh Institute & School of Basic Medicine Zhejiang University School of Medicine Hangzhou 310058 China; ^6^ Department of Gynecology the First Affiliated Hospital School of Medicine Zhejiang University Hangzhou 310058 China; ^7^ The First Affiliated Hospital and Center for Stem Cell and Regenerative Medicine Department of Basic Medical Sciences School of Medicine Zhejiang University Hangzhou 310058 China; ^8^ Edinburgh Medical School University of Edinburgh Edinburgh EH16 4SB UK; ^9^ Department of Sports Medicine School of Medicine Zhejiang University Hangzhou 310058 China

**Keywords:** limb development, lineage tracing‐, musculoskeletal stem cells, single‐cell RNA‐sequencing

## Abstract

Although being of utmost importance for human health and mobility, stem cell identity and hierarchical organization of musculoskeletal progenitors remain largely unexplored. Here, cells from E10.5, E12.5, and E15.5 murine limbs are analyzed by high throughput single‐cell RNA sequencing to illustrate the cellular architecture during limb development. Single‐cell transcriptional profiling demonstrates the identity and differentiation architecture of musculoskeletal stem cells (MSSC), soft and hard tissue progenitors through expression pattern of musculoskeletal markers (scleraxis [*Scx*], *Hoxd13*, *Sox9*, and *Col1a1*). This is confirmed by genetic in vivo lineage tracing. Moreover, single‐cell analyses of Scx knockout mice tissues illustrates that *Scx* regulates MSSC self‐renewal and proliferation potential. A high‐throughput and low‐cost multi‐tissues RNA sequencing strategy further provides evidence that musculoskeletal system tissues, including muscle, bone, meniscus, and cartilage, are all abnormally developed in Scx knockout mice. These results establish the presence of an indispensable limb Scx+Hoxd13+ MSSC population and their differentiation into soft tissue progenitors (*Scx+Col1a1+*) and hard tissue progenitors (*Scx+Sox9+*). Collectively, this study paves the way for systematically decoding the complex molecular mechanisms and cellular programs of musculoskeletal tissues morphogenesis in limb development and regeneration.

## Graphical abstract

This study presents a comprehensive genomics resource that reveals the transcriptome dynamics of the developing limb. Single‐cell transcriptional profiling with lineage tracing data demonstrates the identity and differentiation trajectory of musculoskeletal stem cells into soft and hard tissues. These results emphasize an indispensable role of limb *Scx*+ musculoskeletal stem cell population and provide additional perspective on musculoskeletal biology.

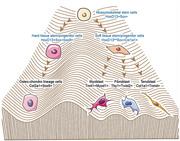

## 1 Introduction

The limb is a complexly patterned, easily observable and experimentally modifiable organ, leading it to be widely used as a model in developmental biology.^[^ ^1^ ^]^ Limb development is a complex morphogenetic process that integrates cells from multiple origins into a well‐organized structure.^[^ ^2^ ^]^ The correct specification of progenitor populations and the ability of these cells to respond to spatiotemporal cues are critical for the successful formation of limbs. Limb malformation is an extremely common type of human malformation that occurs in ≈1 in every 500 births.^[^ ^3^ ^]^ Mechanisms orchestrating limb morphogenesis and differentiation are complex. Currently, there is a lack of sufficient knowledge on the sequential and tightly coordinated cellular events that lead to the establishment of each individual tissue type within the vertebrate limb.^[^ ^4^ ^]^ Increasing understanding of the morphogenesis and cellular origin of the limb will advance our knowledge of how limb malformations occur, as well as provide an important framework for designing better in vitro models and regenerative approaches using stem cell therapies.

Among all the components of an entire limb, musculoskeletal system accounts for a great deal of proportion. In the early stages of musculoskeletal development, each musculoskeletal primordium initially develops as an independent component, and progenitors of musculoskeletal components migrate and settle down in prospective regions to give rise to bone, cartilage, muscle, tendon, and ligament primordia.^[^ ^5^ ^]^ The existence of skeletal stem cells has been identified by lineage tracing and clonal analysis, gradually differentiating into bone, cartilage, and stroma.^[^ ^6^ ^]^ However, the origin of musculoskeletal stem cells (MSSC), which develop into soft and hard connective tissues, have not yet been identified.

Single‐cell RNA sequencing (scRNA‐seq) has emerged as a powerful tool that enables simple and robust access to the transcriptomes of thousands of single‐cells. Recent advances in the single‐cell analysis technologies present great ability to delineate hierarchical cellular states, including intermediate progenitors and the networks of regulatory genes orchestrating cell‐type specification. Given its power to provide comprehensive descriptions of transcriptomic states and their presumptive regulators, scRNA‐seq has been successfully used to phenotypically characterize and classify cells in tens of organs and tissues, as well as to study early lineage diversification.^[^ ^7^, ^8^ ^]^ Recently, the scRNA‐seq work of limb development in the chick and mouse profiled the transcriptional landscape and cell‐type heterogeneity.^[^ ^9^ ^]^ Deeper sequencing at different developmental stages may be necessary to more accurately define the cell population at the transition state and gain insight into transcripts that may have relatively low expression levels. A systematic map of the molecular and cellular dynamics during the early limb development may reveal the characteristic features of limb development and musculoskeletal stem/progenitor cell populations involved in limb lineage development. Herein, we use the Fluidigm C1 system with optimized high‐throughput integrated fluidics circuits (HT IFCs) to carry out single‐cell resolution gene expression profiling during early murine limb development, from E10.5 when the limb bud erupts to E15.5 when most tissue types are specified. We also chose E12.5 as an intermediate developmental stage time point to evaluate the possible stem/progenitor cells during limb morphogenesis. This enables us to successfully create an atlas of gene expression state/patterns in multiple limb cell types, identify MSSC, and characterize the molecular mechanisms controlling early cell lineage decisions in the developing limb.

Genetically modified mice are commonly used for research or as animal models of human diseases. The most common type is the gene knockout mice, where the activity of a single (or in some cases multiple) gene is removed.^[^ ^10^ ^]^ Previous research of whole‐body gene knockout or tissue‐specific knockout mice are often limited to studies of individual organ or phenotype. However, many genes are expressed and function in multiple tissues, and different types of tissues can crosstalk and influence function of the others. Thus, often times, the systematical effects caused by loss of function of the target gene are poorly investigated by examining one tissue type. The unique barcode and index labeling strategy can be used for cost‐effective, organism‐wide transcriptome analysis, which will enable the screening of the most affected tissue or organ due to gene deficiency. Here, using comparative multiple sample RNA‐seq and scRNA‐seq, we successfully generated transcriptome profiles across different tissues in wild‐type and scleraxis (*Scx*) knockout mice, and obtained a cell map of mouse hind limbs at single‐cell resolution.

We also found that *Scx* expression labels a stem/progenitor population that gives rise to musculoskeletal cells of soft tissue (myocytes, meniscus cells, tenocytes) as well as to hard tissue (chondrocytes and osteocytes). Loss of Scx function during limb development leads to a marked decrease in musculoskeletal progenitor expansion and limb patterning defects. Our study demonstrates a strategy for the systematic transcriptome analysis of a gene knockout mouse and provides a blueprint for understanding the function of MSSC on limb development.

## 2 Results

### 2.1 Unbiased Clustering Confirms Known Cell Populations in the Constructed Limb

**Figure 1:**
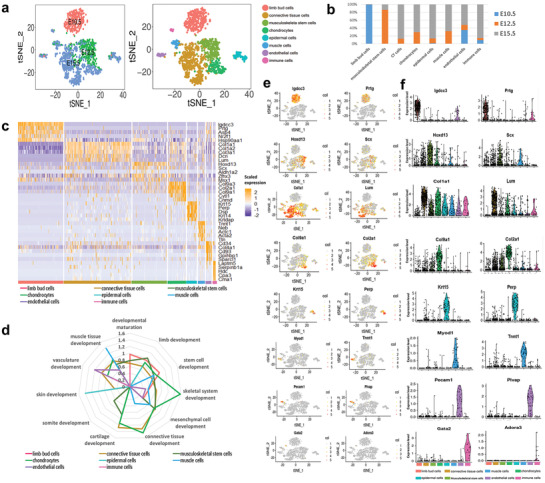
Single‐cell RNA‐seq reveals eight major cell clusters during limb morphogenesis. a) Limb cell transcriptomes visualized with t‐distributed stochastic neighbor embedding (t‐SNE), left panel colored by embryonic stage, and right panel colored according to unsupervised clustering. 1533 single‐cells from mouse hind limb of embryonic (E10.5, E12.5, and E15.5) were grouped into eight distinct clusters (colors indicated). Each point represents an individual cell. b) Component of different stage (E10.5, E12.5, and E15.5) in each cluster during limb development. c) Heatmap of genes significantly enriched within each individual cluster. Single‐cells are shown in columns; genes are shown in rows. Top five differential genes of each cell type are shown. d) The enriched biological processes gene ontology (GO) in different cell clusters. e) Expression of representative cluster‐specific genes projected onto the t‐SNE map. Gray indicates low expression and red indicate high expression. f) Violin plots showing distribution of expression for selected t‐SNE cluster marker genes. Cell types are represented by different colors in (a), (b), (c), and (d).

To determine the cellular composition of the developing limb, we isolated and sequenced transcriptomes of individual live cells from the developing murine hind limb (from the limb bud initiation at E10.5 to a fully patterned hind limb at E15.5) based on Fluidigm C1 system with optimized microfluidic circuits platform, which is the good balance of throughput and resolution.^[^ ^11^ ^]^ After sequencing and data processing, we got high‐quality transcriptomic data from 1533 single‐cells, including 383 E10.5 cells, 383 E12.5 cells, and 765 E15.5 cells. The scRNA‐seq data had high read depth, mapping up to 4000 genes for most of the single‐cell samples (Figure S1, Supporting Information). Our single‐cell transcriptomic data identified eight major cell populations in the developing hind limb by Seurat analysis. Notably, the limb bud cells have relative homogeneity, which was made up of only cells from E10.5 (**Figure** **1**b); E15.5 cells showed significant heterogeneity, which indicated well differentiation of the limb (Figure 1a,b). The heat map showed single‐cell data sets with clear differential gene expression modules and cell‐type clusters (Figure 1c). Other than the limb bud cell cluster, we were able to identify the analyzed six other cell clusters including the connective tissue cells, chondrocytes, endothelial cells, epidermal cells, immune cells, and muscle tissue cells. The other cluster composed of mainly E12.5 cells was assigned as MSSC. Gene ontology (GO) analysis was conducted to gain a better functional insight on different cell clusters; results illustrated that cells in the limb bud cluster highly expessed genes that play key roles in the limb development and developmental maturation (Figure 1d). MSSC cluster was enriched in genes controlling the limb development, stem cell development, skeletal system development, and mesenchymal cell development (Figure 1d), with high expression levels of *Scx* and *Hoxd13*. (Figure 1e,f). *Hoxd13* has been reported to be heterogeneously expressed in different cell types during limb development.^[^ ^9a^ ^]^ The GO terms related to connective tissue development and mesenchymal cell development were highly represented in the connective tissue cells cluster, expressing high levels of *Col1a1* and *Lum* (Figure 1e,f). Additionally, GO terms related to cartilage development and skeletal system development were enriched in the chondrocyte cluster, showing an upregulation of *Col9a1* and *Col2a1* (Figure 1e,f). Remarkably, the endothelial cell cluster was high in genes associate with vasculature development, while GO terms related to skin development were highly represented, specifically in the epidermal cell cluster (Figure 1d). Moreover, the GO term related to immune response was enriched in the immune cell cluster that showed high *Gata2* and *Plvap* expression levels (Figure 1e,f). Cells in the muscle tissue cluster were high in genes controlling muscle tissue development, with high expression levels of *Myod* and *Tnnt1* (Figure 1e,f). We predicted the identity of stem cells using stemID algorithm.^[^ ^12^ ^]^ The inferred lineage tree (Figure S2A, Supporting Information) indicated that limb bud cells (cluster 7) will differentiate into MSSC (cluster 2), which linked to all the other cell clusters including chondrocytes (cluster 3), muscle cells (cluster 5), and connective tissue cells (cluster 1), respectively. Results showed that the *Scx+Hoxd13+* MSSC population exhibited the highest score, which means high degree of multipotency (Figure S2B, Supporting Information).

### 2.2 Identifying Developmental Trajectory and Regulatory Genes during Musculoskeletal System Development

**Figure 2:**
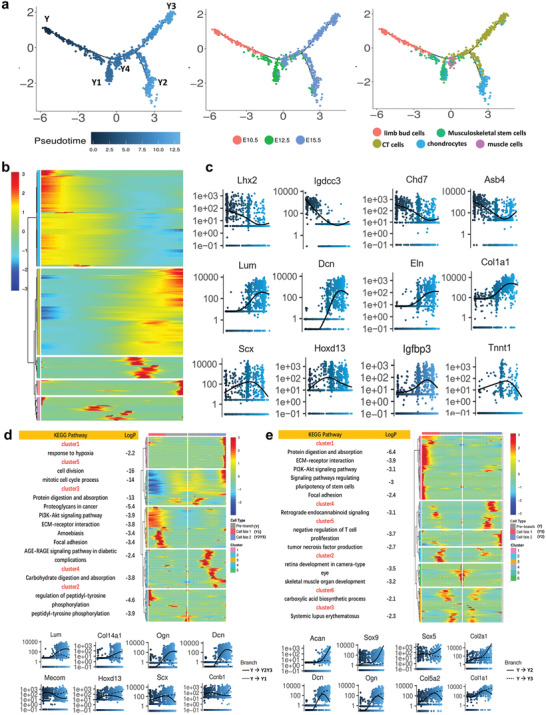
Single‐cell RNA‐seq reveals musculoskeletal tissue cells trajectory during limb morphogenesis. a) Differentiation trajectory of musculoskeletal tissue cells constructed by Monocle. Left panel was colored by pseudotime order. Middle panel was colored by real time point. Right panel was colored by cell clusters. Branches on the 2D trajectory tree are indicated as Y1, Y2, Y3, and Y4. b) Heat map of differentially expressed genes identified by Monocle (rows), with cells (columns) are ordered according to the pseudotime development. c) Plots showing the relative expression of differentially expressed genes in pseudotime order. Each dot represents a single‐cell; different color represents pseudotime order. d) Branch kinetic heatmap of significantly branch‐dependent genes (BEAM test; FDR < 10%) in pseudotime order (upper panel). Branch expression curves for representative significantly branch‐dependent genes (lower panel). Dashed line indicates the spline fit for cells on the path from the root of the tree (Y) in (a) to outcome Y1, while the solid line indicates the curve for the path to Y2Y3. e) Branch kinetic heatmap of significantly branch‐dependent genes (BEAM test; FDR < 10%) at transition point (upper panel). Branch expression curves for representative significantly branch‐dependent genes (lower panel). Dashed line indicates the spline fit for cells on the path from the root of the tree (Y) in (a) to outcome Y3, while the solid line indicates the curve for the path to Y2.

In order to reveal the musculoskeletal system development and sequential waves of regulators that act in differentiation, our further analysis excluded epidermal cell cluster, endothelial cell cluster, and immune cell cluster. Pseudotime ordering of individual cells from the other five clusters were reconstructed by Monocle,^[^ ^8b^ ^]^ an unsupervised algorithm (**Figure** **2**a left panel). The trend of reconstructed trajectory was consistent with the real development time point (Figure 2a middle panel), which could represent the temporal (stem/progenitor and differentiated/maturation lineage) relationships during the development of limb musculoskeletal tissues. The reconstructed trajectory tree colored by clusters shows some overlap along the pseudotime (Figure 2a right panel). The dynamic changes of gene expression along the whole lineage differentiation trajectory are shown in Figure 2b. The repressed genes during differentiation included *Asb4, Lhx2, Chd7*, and *Igdcc3*, while the group of matrix genes such as *Col1a1, Lum, Eln*, and *Dcn* gradually increased along pseudotime progression (Figure 2c). The expression peak of *Hoxd13, Igfbp3, Tnnt1*, and *Scx* were shown in the middle part of pseudotime tree (Figure 2c). As shown in Figure 2a, the hierarchy of musculoskeletal tissue cell lineages was reconstructed into a stepwise developmental cell commitment and differentiation trajectory with four branches. Notably, at the early differentiation point, one branch (Y1) that had high expression levels of *Scx, Mecom, Hoxd13*, and *Ccnb1* gene from the earlier embryonic stage, could be defined as an early stem/progenitor branch, while the other branches strongly expressing matrix genes *Col14a1, Lum, Ogn*, and *Dcn* were defined as a differentiated branch (Figure 2d). We used a specific heatmap to show the gene expression dynamics of these two branches (Figure 2d). There is a separate short branch (Y4) only consisting of cells from muscle cell cluster, which is similar to the development*in vivo* that muscle cells originating from the somite migrate into the limb to complement the formation of musculoskeletal system. At the end of the tree‐like structure, we found two branches following differentiation, the Y2 branch presented the hard tissue differentiation pathway, as evidenced by branch‐dependent expressing high levels of osteochondro‐lineage marker genes *Acan, Col2a1, Sox9*, and *Sox5* (Figure 2e). The Y3 branch expressing high levels of *Dcn, Ogn, Col5a1*, and *Col1a1* might present the other soft tissue differentiation, such as the stromal cell sub‐cluster and the teno‐lineage sub‐cluster (Figure 2e). In summary, the lineage distance showed in this study could represent both the temporal and spatial (hard and soft connective tissues) relationships during the development and maturation of limb musculoskeletal system tissues.

### 2.3 Single‐Cell RNA‐Seq Distinguishes *Scx+* Musculoskeletal Stem Cells during Musculoskeletal System Development

**Figure 3:**
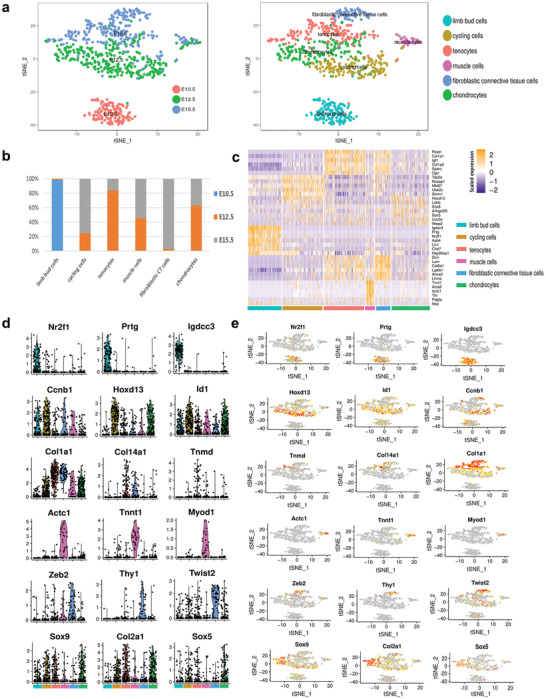
Single‐cell transcriptome analysis distinguishes various cell lineages labeled by *Scx*. a) T‐stochastic neighbor embedding (t‐SNE) plots of *Scx*‐expressing cells during embryonic limb development from Day 10.5 and Day 15.5. Left panel colored by embryonic stage, and right panel colored according to unsupervised clustering. b) Component of different stage (E10.5, E12.5, and E15.5) in each *Scx*‐expressing sub‐cluster. c) A hierarchical clustering heatmap showing differentially expressed genes (row) across *Scx*‐expressing cells (column). Yellow corresponds to a high expression level; white and purple correspond to low expression levels. Top five differential genes of each sub‐cluster are shown. d) Violin plots show the expression level distributions of marker genes across cell types. Cell types are represented by different colors in (a), (c), and (d). e) FeaturePlot of representative genes enriched in each cluster.

Since *Scx* was enriched in MSSC (Figure 1f), its expression is relatively high during the critical stage of fate decision (Figure 2c) before major tissue types are specified. To study heterogeneity lying within *Scx*‐positive MSSC, *Scx*‐expressing cells were included for further analysis; the final scRNA‐seq dataset was comprised of 739 cells. Across all the single‐cells analyzed, 20 577 unique genes were found to be expressed. We performed Seurat analysis and identified six major sub‐clusters of *Scx*‐expressing cells with distinct gene expression patterns (**Figure** **3**c), including limb bud cells, cycling cells, hard tissue lineage cells (osteo‐chondrocytes), and soft tissue lineage cells (tenocytes, muscle cells, and fibroblastic connective tissue cells) (Figure 3a). As expected, all limb bud cells were found to derive from E10.5, while other sub‐clusters were made up of cells from both E12.5 and E15.5 (Figure 3b). Sub‐cluster 4 is annotated as muscle cells due to the high expression levels of *Actc1, Tnnt1* and *Myod1* (Figure 3d,e). Sub‐cluster 6 is annotated as chondrocytes as*Sox9, Col2a1*, and *Sox5* were highly expressed (Figure 3d,e). To further analyze differential gene expression, GO enrichment analysis was performed and representative GO terms in represented biological process were illustrated (Figure S3, Supporting Information). We found that genes specifically expressed in each cell type were enriched in the expected GO terms (Figure S3, Supporting Information). For example, genes that are specifically expressed in muscle cell cluster are significantly enriched in muscle cell development; specific genes of chondrocyte cluster are significantly enriched in skeletal system development and positive regulation of chondrocyte differentiation. These analyses strongly indicated that our cell‐type assignments are accurate. Gene set enrichment analysis showed that cells belonging to sub‐cluster 3 had a relative stronger tendon gene signature and TGF‐beta signaling compared to other sub‐cluster counterparts (Figure S3, Supporting Information).

### 2.4 Lineage Tracing of *Scx*+ Cells in Limb Development Homeostasis

To evaluate the cellular behavior of *Scx*+ cells in limbs, lineage tracing was performed by crossing *Scx‐cre* mice with mice carrying the Rosa26‐mT/mG reporter, which constitutively expresses membrane‐targeted Tomato fluorescentprotein and membrane‐targeted Green Fluorescent Protein (GFP) upon Cre‐mediatedrecombination.^[^ ^13^ ^]^ With this referred to as *Scx*‐cre;Rosa26‐mT/mG mice, live visualization and distinction of recombined *Scx*+ cells (GFP+) and non‐recombined cells are allowed.

**Figure 4:**
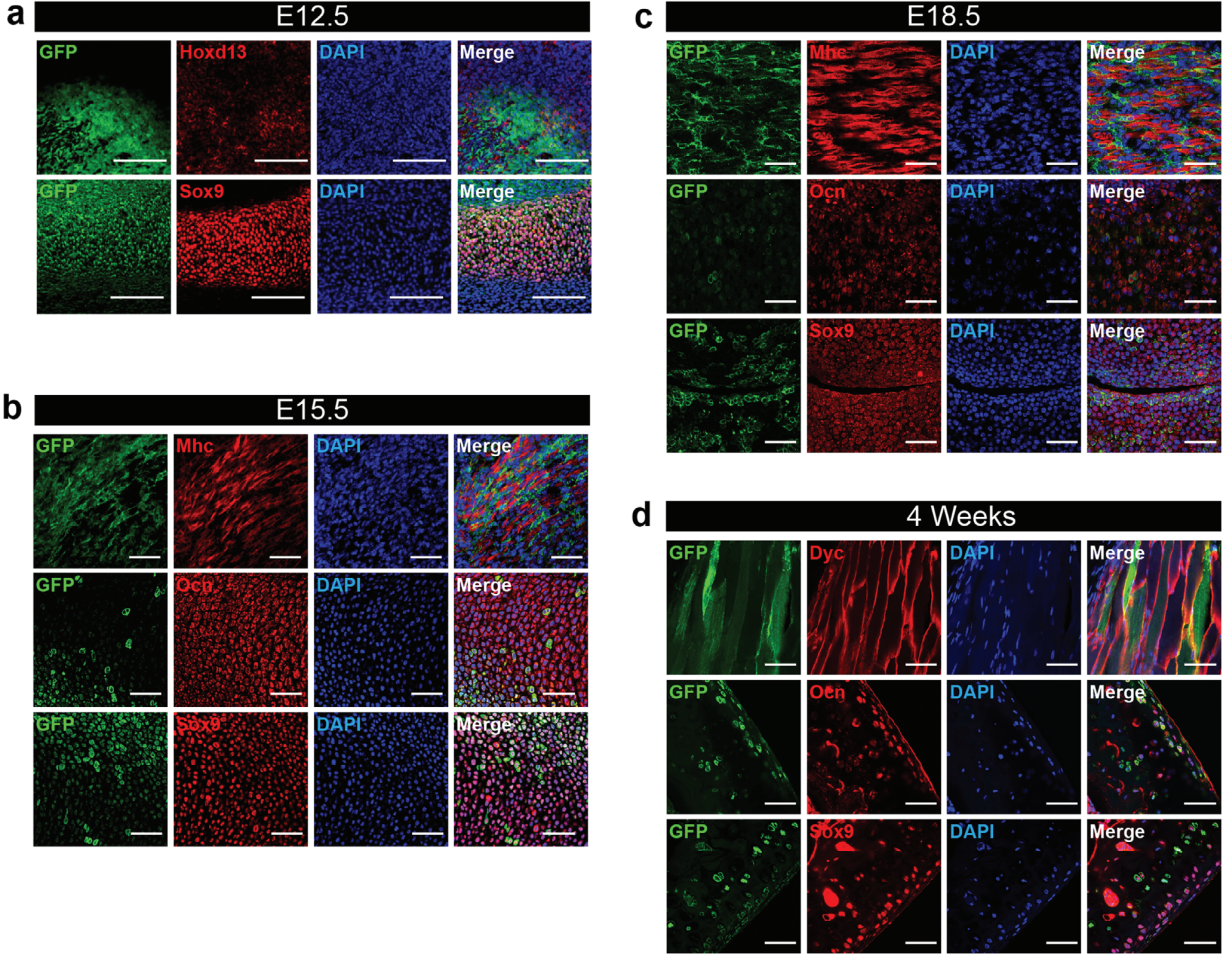
Lineage tracing of *Scx+* cells showed the participation of *Scx*+ cells in musculoskeletal system during limb development. a) Immunofluorescence of Scx (green), Hoxd13, and Sox9 in E12.5 day hind limb. Scale bar, 50 µm. b) Immunofluorescence of Scx (green), Mhc, Ocn, and Sox9 in E15.5 day hind limb. Scale bar, 50 µm. c) Immunofluorescence of Scx (green), Mhc, Ocn, and Sox9 in E18.5 day hind limb. Scale bar, 50 µm. d) Immunofluorescence of Scx (green), Dys, Ocn, and Sox9 in 4‐week hind limb. Scale bar, 50 µm.

To determine whether lineage‐labeled *Scx*+ cells contribute to musculoskeletal system morphogenesis, we detected the existence of lineage‐labeled *Scx*+ cells along with lineage specific markers in multiple tissues during development. As expected, the tendon and ligament tissues at all stages persistently expressed *Scx* (Figure S4, Supporting Information). We validated the musculoskeletal stem cell cluster revealed from scRNA‐seq by evaluating the expression of *Scx* by transgenic labeling and co‐localization with Hoxd13 (**Figure** **4**a). In E12.5 mice, the cartilage primordium of hind limb showed green fluorescence, reflecting the robust expression of this transgene (Figure S3a, Supporting Information). Early cartilage marker Sox9 overlapped with GFP, indicating a chondrogenic potential of *Scx*‐positive cell lineage (Figure 4a). After E15.5, we found that the GFP‐lineage‐labeled cells extended throughout the hind limb, consistent with the hypothesis of *Scx*+ cells being involved in multiple tissue morphogenesis (Figure S4b, Supporting Information). Interestingly, lineage‐labeled *Scx*‐derived GFP+ cells are located in the myofiber interstitium, but neither in the myofiber nor co‐stained by Mhc at E15.5 and E18.5 (Figure 4b,c), which is similar to other non‐satellite cells muscle progenitors.^[^ ^14^ ^]^ At day E15.5 and E18.5, immunofluorescence staining revealed that there were *Scx*‐mG‐labeled cells co‐expressing osteo‐lineage marker Ocn, or cartilage marker Sox9 (Figure 4b,c). To further investigate whether *Scx*+ cells take part in the formation of musculoskeletal system besides tendon in the adult stage, limbs were harvested over 4‐week period and sections were analyzed with confocal microscopy for the presence of lineage‐labeled cells. Notably, lineage‐labeled cells could still be observed within the musculoskeletal system, indicating that *Scx*‐lineage cells could contribute to musculoskeletal lineages during homeostasis (Figure 4d). Furthermore, we purified the *Scx*‐mG+ cells from *Scx*‐cre;Rosa26‐mT/mG mice limb bud at E10.5. Then the cells were injected into a wild‐type developing mouse limb bud at E15.5, then transplanted subcutaneously into nude mice. At 2 weeks, immunofluorescence staining revealed there were *Scx*‐mG‐labeled cells co‐expressing myofiber marker Myh3, or connective tissue matrix Col1, or chondrogenic marker Sox9, or osteogenic marker Runx2 (Figure S5, Supporting Information). Thus, *Scx*‐mG+ labeled cells in the musculoskeletal system showed characteristics of stem/progenitor cells.

### 2.5 Resolving Cellular Heterogeneity of WT and *Scx^−/−^* Mouse Limbs during Development by Single‐Cell RNA‐Seq

Expression of *Scx* was upregulated at the onset of both fore limb and hind limb formation since E10.5 and persisted until E12.5, tapering off at later time point (Figure S6, Supporting Information). Thus, we reasoned that the investigation of developing limbs from wild‐type and *Scx^−/−^* mice by scRNA‐seq could provide insights into the cellular mechanisms underlying musculoskeletal dysplasia.

To obtain cells for scRNA‐seq analysis, hind limbs of E12.5 embryos were carefully isolated under microscopy and enzymatically dissociated; at the same time, the tails were isolated for genotyping. Wild‐type and *Scx^−/−^* embryos were used for scRNA‐seq. After quality control, we retained 761 individual cells, including 372 wild‐type and 389 *Scx^−/−^* cells for the subsequent analysis. The average number of genes expressed in each wild‐type cell and *Scx^−/−^* cell were 2723 and 4051, respectively.

**Figure 5:**
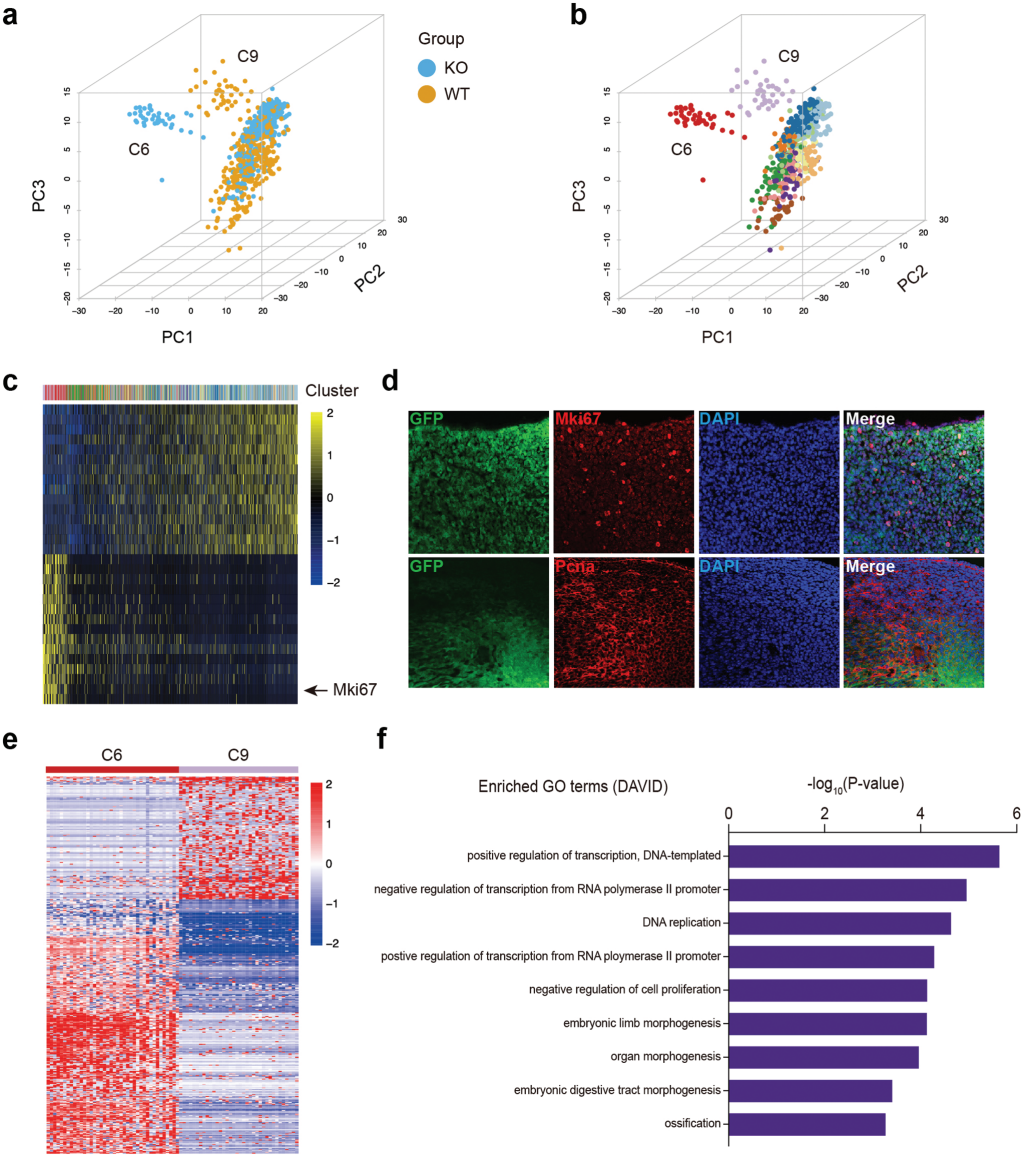
Single‐cell RNA‐seq analysis of hind limbs from embryonic wild‐type and *Scx^−/−^* mice. a) A 3D PCA of single‐cell RNA‐seq data for E12.5 mice hind limb cells. Plots are colored by genotypes. b) A 3D PCA of single‐cell RNA‐seq data for E12.5 mice hind limb cells. Plots are colored by cell types. c) A PC heatmap showing sources of heterogeneity PC1 in E12.5 mice hind limb single‐cell RNA‐seq data. Cells and genes are sorted by their principal component scores. d) Immunofluorescence of Scx‐GFP, Mki67, and Pcna in E12.5 day hind limb. Scale bar, 50 µm. e) A gene expression heatmap showing differentially expressed genes for cell cluster C6 and C9 in E12.5 mice hind limb single‐cell RNA‐seq data. Red corresponds to high expression level; Blue and white correspond to low expression levels. f) The enriched GO terms (biological processes) of differentially expressed genes for cell cluster C6 and C9. Up, GO terms of genes upregulated in C9; down, GO terms of genes downregulated in C9.

We performed a cell clustering analysis based on high variable gene. Identification of genes significantly enriched in each cluster highlighted common features within these clusters. GO analysis was then performed based on genes significantly enriched in each cluster to gain a functional insight of each cell cluster. In general, most of the cells can be classified into immune cells, and tissue stem/progenitor cells according to their gene expression patterns and functional characteristics (Figure S7, Supporting Information). Interestingly, according to the 3D‐PCA plot (**Figure** **5**a), cluster C6 and C9 were distinct from other cell clusters on PC1; it is intriguing that all cells in cluster C6 were *Scx^−/−^* cells and all cells in cluster C9 were wild‐type cells, indicating that the distinct gene expression pattern between these cell clusters might exert different influence on the development of musculoskeletal tissues (Figure 5b).

We then looked to find out the differences between these cell clusters and other cell clusters, as well as their intricate differences. First, we examined the gene expression pattern on PC1, which distinguishes C6 and C9 from other cell clusters. In consistent with the result above, we found that *Mki67* was highly expressed in C6 and C9, indicating that these cells were proliferative cells (Figure 5c). To assess the proliferative potential of *Scx*+ cells, proliferative markers were evaluated. Tissues from E12.5 Scx‐cre; Rosa26‐mT/mG mice hind limbs were harvested and we detected GFP+ cells contained cells co‐expressing proliferation markers (Mki67 and Pcna, Figure 5d). Next, we identified differentially expressed genes (DEGs) between cell cluster C6 and C9 (Figure 5e). GO analysis showed that genes related with embryonic limb morphogenesis, organ morphogenesis, and ossification, were downregulated in C6 (Figure 5f). These results demonstrated the loss of *Scx^−/−^* altered expression pattern of genes related to limb morphogenesis in a highly proliferative progenitor cell population during limb development.

### 2.6 Organism‐Wide RNA‐Seq Analysis of 16 Tissues in Wild‐Type and *Scx^−/−^* Mice

According to the Human Protein Atlas (www.proteinatlas.org) and Mouse Genome Informatics Database (www.informatics.jax.org),^[^ ^15^ ^]^ expression of *Scx* can be detected in diverse tissue types, suggesting that *Scx* deficiency could result in organism‐wide effects. To perform a comprehensive transcriptomic profiling of different tissues in wild‐type and *Scx^−/−^* mice (Figure S8a, Supporting Information), we introduced a multiplexed RNA‐sequencing (MuSeq) strategy. Different tissue samples were labeled with unique barcodes and reverse transcribed, and then pooled to construct cDNA library for sequencing as described before (Figure S8b, Supporting Information).^[^ ^16^ ^]^

By MuSeq, we embarked on obtaining transcriptome profiles of all major organs or tissues in mice. We analyzed adipose, bladder, bone, cartilage, cortex, heart, hippocampus, intestine, kidney, liver, lung, muscle, rectum, spleen, stomach, and vascular from wild‐type and *Scx* knockout mice. In general, the number of genes detected in each tissue varied from each other, and an average of 8426 genes were detected across all samples. To obtain an overview of the power of MuSeq to recapture inter‐sample differences, a t‐distributed stochastic neighbor embedding (t‐SNE) analysis was performed for dimensionality reduction and data visualization. A t‐SNE map demonstrats that most tissues can be clearly separated except for the cortex and hippocampus, possibly due to their similarity in transcriptional profile (Figure S8c, Supporting Information, showed).

### 2.7 Tissue‐Specific Genes Reflect Tissue Biological Functions

We then looked to identify tissue‐specific genes (TSGs) that were highly expressed and relatively specific to each tissue. To identify TSGs, we introduced a method which has been developed for identifying cell‐type marker genes.^[^ ^17^ ^]^ We calculated the area under the receiver operating characteristic (AUROC) curve to quantify the accuracy of the prediction, and a *p*‐value was assigned to each gene using the Kruskal–Wallis test, comparing gene ranks in the tissue with the highest mean expression with all others. Genes with AUROC ＞ 0.9 and with *p* ＜ 0.001 were defined as TSGs. The number of TSGs identified in the wild‐type and *Scx^−/−^* tissues ranged from 17 to 1041 and 13 to 1108, respectively (Figure S8d, Supporting Information).

We performed GO enrichment analysis of TSGs in each tissue type. In general, the enriched GO terms based on TSGs were highly consistent with the biological functional activities of the tissue for which the genes were enriched. For example, wild‐type cortex‐specific genes were active in biological functions related to diverse neurophysiological processes, including chemical synaptic transmission and nervous system development, whereas the biological function enrichment pattern for spleen‐specific genes included various immunological processes such as immune system process, adaptive immune response, and B cell activation. However, both wild‐type and *Scx^−/−^* vascular‐specific genes were related to brown fat cell differentiation, indicating the contamination of vascular samples; we thus excluded this tissue for the following analysis.

### 2.8 Differential Expression Patterns Reflect Functional Differences between Wild‐Type and *Scx^−/−^* Musculoskeletal Tissues

**Figure 6:**
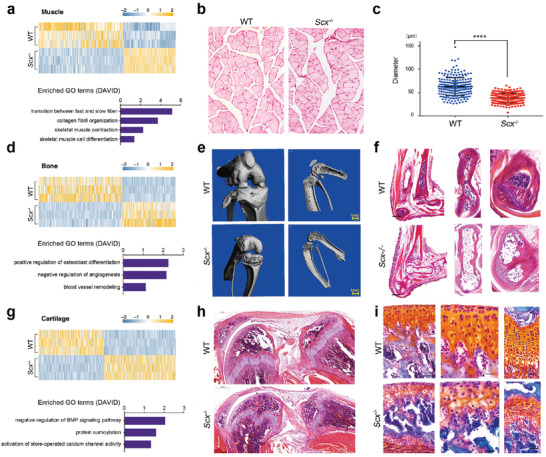
*Scx* deficiency results in abnormal development of musculoskeletal system tissues. a) A gene expression heatmap showing differentially expressed genes (FC ＞ 2 and *p* ＜ 0.05) between wild‐type and *Scx^−/−^* muscles. The enriched GO terms (biological processes) of genes downregulated in *Scx^−/−^* muscles are shown below. b) H&E staining of the muscles in 3‐week‐old wild‐type and *Scx^−/−^* mice. Scale bar, 50 µm. c) Statistical analysis of average diameter of muscle fibers. n(WT) = 226, n(*Scx^−/−^*) = 213. Unpaired *t*‐test, *p*  ＜0.0001. d) A gene expression heatmap showing differentially expressed genes (FC ＞ 2 and *p* ＜ 0.05) between wild‐type and *Scx^−/−^* bones and corresponding enriched biological process terms of DAVID gene ontology analysis. The enriched GO terms (biological processes) of genes downregulated in *Scx^−/−^* bones are shown below. e) MicroCT examination of knee joints from wild‐type and *Scx^−/−^* mice. Scale bar, 1 mm. f) H&E staining of the astragalus and calcaneus in wild‐type and *Scx^−/−^* mice. g) A gene expression heatmap showing differentially expressed genes (FC ＞ 2 and *p* ＜ 0.05) between wild‐type and *Scx^−/−^* cartilages and corresponding enriched biological process terms of DAVID gene ontology analysis. The enriched GO terms (biological processes) of genes downregulated in *Scx^−/−^* cartilages are shown below. h) H&E staining of the knee joint in wild‐type and *Scx^−/−^* mice. Scale bar, 500 µm. i) Safranin O‐fast green staining of the joint cartilages and meniscus in 3‐week‐old wild‐type and *Scx^−/−^* mice. Scale bar, 100 µm.

To uncover the effects of *Scx* deficiency on the gene expression pattern of each tissue, we used DESeq2 to identify genes that were differentially expressed (*p*‐value  ＜0.05, fold change (FC)  ＞2 or ＜−2) between any tissue in *Scx^−/−^* mice and its counterpart in wild‐type mice.^[^ ^18^ ^]^ Although GO enrichment analysis of DEGs revealed that many of the Scx regulated genes were not associated with tissue‐specific biological functions, we identified consistent downregulation of functional important genes in musculoskeletal tissues. For example, some genes pointed to skeletal muscle contraction (*Tnnt1, Atp2a2, Tnnc1*, and *Tnni1*) and collagen fibril organization (*Fmod, Col1a2, Col2a1, Col1a1*, and *Serpinh1*) were downregulated in *Scx^−/−^* muscles (**Figure** **6**a), reflecting in the significantly smaller muscle fiber diameter (Figure 6b,c). Similarly, genes related with collagen fibril organization, such as*Col1a1*, *Col1a2*, and*Serpinh1* were also downregulated in *Scx^−/−^* cardiac tissue. The expression of *Tsp4*, a myocyte‐interstitial mechano‐signaling molecule central to adaptive cardiac contractile responses to acute stress, was also suppressed in *Scx^−/−^* cardiac tissue. In addition, genes known as markers for the positive regulation of osteoblast differentiation, such as *Cebpb, Vegfa, Jag1, Bmpr1b*, and *Gdpd2*, were downregulated in bones upon Scx deficiency (Figure 6d), which mirrored the defects in bone detected by micro‐CT (Figure 6e). The trabecular bone mass in the astragalus and calcaneus of *Scx* KO mice was significantly reduced, and its shape appeared to be irregular (Figure 6f). For the cartilage, we identified downregulated genes related to the negative regulation of transforming growth factor beta receptor (Tgfbr) and BMP signaling pathway (Figure 6g). The regulation of Tgfbr and BMP signaling is important in chondrocyte terminal differentiation and cartilage maturation.^[^ ^19^ ^]^ H&E and Safranin O‐fast green staining showed that *Scx* knockout cartilage was less mature than wild‐type (Figure 6 h,i). Taken together, analysis of organism‐wide transcriptomic data identified that musculoskeletal tissues were the most affected tissues due to *Scx* deficiency.

### 2.9 *Scx*+ Cell Contribute to Limb Endogenous Repair and *Scx*+ Cell Ablation Impairs Limb Injury Repair

We used *Scx‐GFP* mice to study the distribution and expression level of *Scx*‐positive cells in tissue repair process (Figure S9, Supporting Information). From the IF staining, we observed that *Scx*‐positive cells were expressed in the injured articular cartilage, meniscus, Achilles tendon, tibialis anterior muscles, and fibula tissue. When compared to the control group, more positive cells were observed to gather at the injury site suggesting that *Scx*+ cells were involved in the repair of limb injuries.

To investigate the role of *Scx*‐expressing cells in the limb repair, we selectively ablated *Scx*+ cells *in vivo* using the *Scx*‐cre‐diphtheria toxin receptor (DTR) mouse model (Figure S10a, Supporting Information). We crossed mice with an inducible human DTR (iDTR) to*Scx*‐cre mice; in this system, musculoskeletal tissues injury models were made separately, subsequently following ablation of *Scx*+ cells by DT treatment. For the articular cartilage injury model, the control group showed fibrous tissue and cartilage‐like tissue formed at the injury site. While, lesions in the *Scx* scavenging group had obvious depressions, the surface layer was covered with a large amount of fibrous tissue, and the matrix was arranged disorderly (Figure S10b, Supporting Information). Thus, these results indicate that ablation of*Scx*+ cells hindered the repair of damaged cartilage tissue.

In the meniscus injury model, H&E staining results showed that the control group contained a large number of fibrochondrocytes, while the *Scx*+ cells scavenging group only had a small number of fibrochondrocytes around the damaged tissue, and the anterior horn of the meniscus was incomplete. From the safranin O staining, a large amount of red‐stained cartilage matrix was found in the control group, while the cartilage matrix in the *Scx*+ cells scavenging group was scattered in the cartilage lacuna and chondrocytes (Figure S9c, Supporting Information). From these results, it can be seen that ablation of *Scx*+ cells reduces the production of fibrochondrocytes in the meniscus and thus delays meniscus tissue repair.

In the *Scx*+ cells scavenging group had a large number of inflammatory cell infiltration at the tendon injury site; the collagen fibers in the scar tissue were disorderly arranged and the cartilage matrix was widely expressed. In the control group, the GAG deposition was mainly at the edge of the injury (Figure S10d, Supporting Information). This shows that the ablation of *Scx*+ cells leads to insufficient repair after tendon injury, and *Scx* plays an indispensable role in healing of adult tendon injury.

Quantitative analysis of the myofiber cross‐sectional area (CSA) showed that the CSA area of the *Scx* scavenging group was significantly smaller than the control group (Figure S10e, Supporting Information). These results indicate that ablation of *Scx*+ cells will affect the muscle regeneration process.

From the H&E staining and Massons's trichrome staining, we found that the control group had a large amount of cancellous bone at the broken end of the fracture. While, there were a lot of hypertrophic chondrocytes and less bone matrix around the broken end of the fracture in *Scx*+ cells scavenging group (Figure S10f, Supporting Information). These results showed that the ablation of *Scx*+ cells can delay the formation of mineralized bone tissue, and affect the regeneration of bone tissue.

## 3 Discussion

Single‐cell transcriptomic analysis has been increasingly applied to the study of molecular eventsand subpopulation functions of various cells/tissues, particularly rare stem/progenitor cells due to its ability to reveal cell heterogeneity. Our study has provided an scRNA‐seq resource, and the complete and dynamic repertoire of transcriptome underlying murine limb development in vivo. We have further identified novel cell subpopulations during limb morphogenesis, especially the *Scx+ Hoxd13+* musculoskeletal stem/progenitor cells. In this study, scRNA‐seq analysis of the developing limb provides new insights into the musculoskeletal tissue differentiation trajectory and uncovers sequential waves of expression of regulators that act in differentiation. Hierarchical and pseudotime ordering identified expression profiles that are likely to correlate with functional musculoskeletal tissue morphogenesis programs during limb development. The branched trajectory indicated that a precursor population exists in early stage of musculoskeletal system development, and in the late stage hard and soft connective tissue formed distinct branches. Our data will help to better understand limb development at the cellular and molecular levels.

Understanding the transcriptome is essential for revealing the molecular constituents of cells and tissues and interpreting the function of a gene. The profiling of gene expression is a prevailing approach to reveal the characteristics of the transcriptional machinery between different status of tissues and cells. RNA sequencing (RNA‐seq) is a revolutionary tool for transcriptome profiling that uses next generation sequencing technologies.^[^ ^20^ ^]^ However, the high cost of RNA‐seq limits its application for organism‐wide transcriptome analysis. Here, we introduced a multiple sample RNA‐seq strategy (MuSeq), a more cost‐effective strategy, and constructed an organism‐wide transcriptome map of wild‐type and *Scx^−/−^* mice. A set of barcodes can be used to label tens of RNA samples, followed by sample pooling for library preparation and high‐throughput sequencing. The cost of sequencing for each sample is estimated to be 10–20 USD depending on the depth of sequencing. As exemplified by our study, MuSeq is applicable to nearly all tissue types. It is an efficient and inexpensive multiple sample RNA‐seq method. However, it is necessary to mention that this strategy is a 3′ end enriched RNA‐seq, which misses the RNA molecules with no ploy‐A tails and limits the downstream analysis such as alternative splicing analysis. Using MuSeq, we profiled gene expression of 16 non‐sexual solid tissues and successfully captured the transcriptome signatures of different tissues. GO analysis indicated that TSGs were well correlated with the functional characteristics of each tissue. In addition, analysis of DEGs demonstrated that the loss of *Scx* may exert different effects on different tissues. Due to space limitations, here, we only presented results for the most effected tissues in the musculoskeletal system including bone, cartilage, and muscle according to the results of MuSeq and histological examination, which suggested that *Scx* plays a role in not only tendon tissue formation but also in other musculoskeletal tissues morphogenesis.

Normally limb musculature is derived from dorsolateral cells of the somites that migrate into the limb to form muscles. Meanwhile, non‐satellite cell muscle resident progenitors were identified to partially contribute to the muscle development and homeostasis repair.^[^ ^14^ ^]^ The mesoderm of the early limb bud comes from the lateral plate mesoderm. The lateral plate mesoderm cells give rise to the limb connective tissues cartilage, bone, tendon, and muscle connective tissue. This study suggests the possibility of multipotent MSSC contributing to both the connective tissue and partialmuscle tissue development and repair.

The tendon progenitor cell population is derived from the syndetome, lateral plate mesoderm, and neural crest.^[^ ^2^ ^]^ The syndetome is a *Scx*‐positive subdomain that occupies the dorsolateral portion of the sclerotome to form the axial skeleton.^[^ ^21^ ^]^ *Scx* also marks the progenitor cells of tendons and ligaments in the appendicular and craniofacial regions.^[^ ^22^ ^]^ *Scx* is a member of the basic helix‐loop‐helix family of transcription factors and is critical for the proper development and maturation of tendons and ligament.^[^ ^22^, ^23^ ^]^ In *Scx*−/− mice, force‐transmitting and inter‐muscular tendons are reported to be affected and are hypoplastic, whereas the muscle‐anchoring tendons and the ligaments are not affected.^[^ ^24^ ^]^ Notably, the size and weight of *Scx* ^−/−^ mice are much smaller than wild type mice, and *Scx* ^*−*/−^ mouse showed drastic limitations of motion, indicating an organism‐wide influence of *Scx* deficiency.^[^ ^23b,d^ ^]^ Recent studies demonstrated that *Scx*‐lineage mesenchymal cells within muscle and tendon contribute to the pathologic development of heterotopic ossification.^[^ ^25^ ^]^ Loss of*Scx* in mice leads to geometric and structural changes in cortical bone, as well as asymmetry in fracture healing.^[^ ^26^ ^]^ Recently, it has been reported that *Scx* is also important for the proper integration of musculoskeletal components, although its expression occurs transiently in the chondrogenic cell lineage and entheseal cartilage.^[^ ^27^ ^]^ Although these efforts have greatly advanced our knowledge of the wide expression of *Scx*, many open questions remain with respect to the identity and regulation of the early migration and specification events of *S* *cx*‐lineage cells. By using scRNA‐seq and lineage tracing, we revealed a previously unrecognized population of musculoskeletal stem/progenitor cells marked by the expression of*Scx*. The findings of this single‐cell analysis improved the resolution of gene functions at cell subpopulation level rather than tissue level.

*Scx*+ cells displayed a unique gene expression profile and contributed to not only tenocytes, but also myocytes, chondrocytes, and osteocytes. According to our evidence, *Scx*+ cells have the potential to contribute to both soft and hard tissue in the musculoskeletal system of the hind limb during development. This universality remained even in adult tissues homeostasis. We found that *Scx, Mecom, Hoxd13*, and *Ccnb1* gene, which from earlier embryonic stage, could be defined as an early progenitor branch. The Y2 branch presented the hard CT differentiation pathway with high levels of osteochondro‐lineage marker genes *Acan, Col2a1, Sox9*, and *Sox5*. The Y3 branch expressed high levels of *Dcn, Ogn, Col5a1*, and *Col1a1*, presenting the other soft CT. Genetic knockout experiments revealed that *Scx*+ cells are required for the development of muscle tissue, osteo‐cartilage lineage, and tendon formation, which is consistent with a previous report stating that *Scx* is required for the proper integration and development of musculoskeletal system in zebra fish.^[^ ^28^ ^]^ The deletion of *Scx* resulted in the loss of *Scx+ Hoxd13+* cell population, which shared overlapping cell proliferative marker profiles (e.g., *Pcna* and *Mki67*) indicating a stem cell fate potential. Taken together, the continuous participation of *Scx*+ cells in musculoskeletal system development lead us to characterize a *Scx*‐lineage cell population in cellular and molecular level and determine its subset of soft and hard tissue precursors responsible for musculoskeletal tissues. This finding renewed current knowledge about *Scx*+ progenitor cells and their functions (Figure S11, Supporting Information).

The current study using single‐cell technologies shed new light on novel musculoskeletal subpopulations and prospective markers for their isolation, which facilitated the proper seed cells selection in translational research. The extracellular matrix properties and dynamic signaling molecules during the lineage specification were revealed by trajectory reconstruction based on the transcriptional landscape of murine hind limb development. This information may be used in *in vitro* systems to more robustly drive embryonic, mesenchymal, or induced pluripotent cells to musculoskeletal tissues for tissue engineering applications. Additionally, a thorough understanding of the limb development process will hopefully allow recapitulation of complex tissue level phenotypes and sequential cytokine signal in controlled culture environments for organoid construction. The precise sequence of events involved in limb development may provide new targets to stimulate regeneration of musculoskeletal tissues.

## 4 Conclusion

In summary, our findings have revealed the diversity of limb cell types, providing a valuable resource for further investigations of the molecular mechanisms regulating limb development. Our study has also allowed the identification of a muscloskeletal stem/progenitor cell population labeled by transcription factor *Scx*, which contribute to the development of the musculoskeletal tissues.

## 5 Experimental Section

##### Animals and Tissue Sampling

Rosa26‐mT/mG (TdTomato‐GFP) mice^[^ ^13^ ^]^ were obtained from Nanjing BioMedical Research Institute of Nanjing University (NBRI) and crossed with Scleraxis‐cre mice kindly provided by Dr. R. Schweitzer (Oregon Health & Science University, Oregon, USA). Rosa26‐stop‐iDTR (DTR) mice were kindly provided by Dr. Wen‐biao Gan (Peking University Shenzhen Graduate School). All animal studies had ethical approval from the Institutional Animal Care and Use Committee of Zhejiang University (#ZJU20170006). Sixteen organs or tissues, including adipose, bladder, bone, cartilage, cortex, heart, hippocampus, intestine, kidney, liver, lung, muscle, rectum, spleen, stomach, and vascular from 3‐week‐old wild‐type and *Scx^−/−^*mice were used in this study. At necropsy, whole organs were removed, quick‐frozen in liquid N_2_, and stored at −80 °C for RNA extraction, or fixed in 4% paraformaldehyde for histological analysis.

##### RNA‐Seq Experiments

RNA‐seq was modified from a previous method. In brief, total RNA was extracted from tissue samples using trizol reagent (TaKaRa); reverse transcription was conducted by SuperScript II reverse transcriptase (Invitrogen); double strand cDNA was conducted using NEBNext mRNA second strand synthesis kit (NEB); double strand DNA was cleaned with AMPure XP beads (Beckman Coulter); sequencing library was constructed with Nextera XT kit (Illumina) and sequenced on Illumina X Ten platform. Sequence reads were mapped to reference genome mm10 using Bowtie2 using default parameters and per gene counts were calculated using HTSeq.^[^ ^29^ ^]^

##### RNA‐Seq Data Analysis

All the statistical analyses were conducted using R statistical programming language. Digital expression data was converted to counts per million by diving with the total number of reads and multiplying by 10^6^. Hierarchical clustering analysis was performed using Ward linkage based on a distance matrix of the Pearson correlation of the samples, and t‐SNE analysis was performed using Rtsne package. To identify TSGs, the area under the ROC curve was first calculated to quantify the accuracy of the prediction, and a *p*‐value is assigned to each gene using the Kruskal–Wallis test, comparing gene ranks in the tissue with the highest mean expression with all others. Genes with areas under the ROC curve (AUROC)  ＞0.9 and with *p*  ＜0.001 were defined as TSGs. DESeq2 was used to identify DEGs between each tissue from wild‐type and *Scx^−/−^* mice.^[^ ^18^ ^]^ In this analysis, a gene was considered to be expressed in a sample if its count value was equal or greater than one in the sample. Genes with count values of zero across all samples were removed. DEGs were defined as FC  ＞2 and *p*‐value  ＜0.05. GO analysis was performed using DAVID.^[^ ^30^ ^]^

##### Preparation of Single‐Cell Suspension from Mice Limb

Single‐cell experiments were performed on the embryonic Scx‐GFP mouse hind limb at E10.5, E12.5, and E15.5, also on the hind limbs from E12.5 day wild‐type and *Scx^−/−^* mice. In general, embryonic experiments were performed on pooled sibling limbs of one litter (three to five limbs per pool). Embryos were genotyped and wild‐type and *Scx^−/−^* mice were dissected and limbs were isolated. The authors finely chopped the tissue with small scissors in a drop of Dulbecco's modified Eagle's medium (DMEM), and then digested the tissues in type I collagenase 0.1%/trypsin 0.05% (Life Technologies) diluted in low‐glucose DMEM (L‐DMEM) (Gibco) solution at 37 °C for 10 min. Then, the tissues were let to settle down and the supernatant was collected in a fresh 50 mL tube, before adding 10% of fetal bovine serum to quench trypsin (on ice to stop collagenase). The digestion steps were repeated for four times or more till all tissues were digested. The supernatants were then pooled. The pooled supernatants were filtered through 70 µm cell filters (Falcon BD), and centrifuged for 10 min at 1600 rpm, before re‐suspending the pellet in DMEM containing 2% serum, and the cell suspension was kept on ice until load on chip.

##### Single‐Cell Capture, cDNA Library Preparation and Sequencing

Single‐cell capture, RNA extraction, and cDNA preparation were performed following the methods described in the Fluidigm protocol (PN 1009886, Using the C1 HT IFC to Generate Single‐Cell cDNA Libraries for mRNA Sequencing). In detail, cells were loaded onto the chip at a concentration of 300 500 cells uL^−1^, and imaged by phase‐contrast microscopy to assess the number of cells per capture site. Wells with more than one cell were excluded for the following analysis. The cDNA reaction products were quantified using the Qubit and were then diluted to a final concentration of 0.2 ng µL^−1^ using C1 Harvest Reagent. The diluted cDNA reaction products were then converted into mRNA‐seq libraries using the Nextera XT DNA sample preparation kit (Illumina, FC‐131‐1096, ‐2001 and ‐2002) following the manufacturer's instructions. The qualified library was then sequenced on Illumina Hiseq X Ten.

##### Processing of the scRNA‐Seq Data

Raw sequencing reads was processed with Perl scripts to ensure the quality of data used in further analysis. The authors first removed the adaptor‐polluted reads (reads containing more than five adapter‐polluted bases) and the low‐quality reads (reads with the number of Phred quality value less than 19 accounting for more than 15% of total bases) and then discarded reads with number of N bases accounting for more than 5%. The obtained clean data was mapped to the mm10 genome release with Bowtie2 using default parameters. Reads count for each gene in each sample was counted by HTSeq. After obtaining the digital gene expression data matrix, The authors used Seurat for dimension reduction, clustering, and differential gene expression analysis.^[^ ^31^ ^]^ For quality control, The authors excluded cells in which less than 2000 genes or more than 8000 genes were detected and genes that are detected in less than 10 cells. This resulted in 1533 cells expressing a total of 20 050 genes for E10.5, E12.5 day, and E15.5 hind limb data, and 761 cells expressing a total of 21 616 genes for E12.5 day wild‐type and *Scx^−/−^* mice data. Dimensional reduction was performed and cell clusters were identified based on the most significant principle components. Genes significantly enriched in each cell cluster were identified using the default algorithm in Seurat. Functional annotation of the resulting marker gene lists relative to GO terms was performed using DAVID. Trajectory analysis of the CT development was performed using Monocle (v2.4.0).

##### Histology and Immunofluorescence

Embryo limbs were harvested at desired ages, and fixed in 4% paraformaldehyde for 1 day at room temperature, then switched to 30% sucrose/PBS overnight before they were frozen, embedded, and sectioned. Adult tissues were harvested at desired ages, and fixed in 4% paraformaldehyde for 1 day at room temperature, then decalcified in 10% w/v EDTA (pH 7.4) for 20 days before the joints were embedded in paraffin. Sagittal joint sections were processed for H&E and safranin O‐fast green staining. For immunofluorescence, the following antibodies were used: GFP (Beyotime, AG281; Cell Signaling Technology, 255S), osteocalcin (Millipore, AB10911), Sox9 (Abcam, ab76997), dystrophin (Abcam, ab15277), Mhc (DSHB, MF‐20), Myh3 (DSHB, F1.652), and Runx2 (Sigma, HPA022040); secondary antibodies conjugated with Alexa Fluor 488 or Alexa Fluor 546 or Alexa Fluor 647 or Alexa Fluor 594 fluorescent dyes (Thermo Fisher Scientific) were used for immunofluorescent staining. The stained specimens were photographed digitally under confocal microscope (Nikon A1R).

##### Statistical Analysis

One‐way ANOVA and Student's *t*‐test were performed to assess whether there were statistically significant differences in the results between groups. Values of *p* < 0.05 were considered to be significantly different. The significance level was presented as *p < 0.05.

## Conflict of Interest

The authors declare no conflict of interest.

## Author Contributions

Z.Y. and J.L. contributed equally to this work. Conceptualization — X.C., X.Z., and H.W.O.; Supervision — X.C. and H.W.O.; Investigation — Z.Y., J.L., W.Z., M.L., R.L., B.Z., Y.C., and Y.H.; Formal Analysis — J.L. and R.Y.; Methodology — K.Z., C.F., and C.A.; Writing–Original Draft — Z.Y. and J.L.; Writing–Review & Editing — A.E., J.Z., and H.W.O.; Funding Acquisition — X.C., B.W., and H.W.; All authors read and approved the manuscript.

## Supporting information

Supporting Information

## References

[1] F. Petit , K. E. Sears , N. Ahituv , Nat. Rev. Genet. 2017, 18, 245. 28163321 10.1038/nrg.2016.167

[2] A. E. Brent , C. J. Tabin , Curr. Opin. Genet. Dev. 2002, 12, 548. 12200160 10.1016/s0959-437x(02)00339-8

[3] J. E. VanderMeer , N. Ahituv , Dev. Dyn. 2011, 240, 920. 21509892 10.1002/dvdy.22535 PMC3174732

[4] A. Zuniga , Development 2015, 142, 3810. 26577204 10.1242/dev.125757

[5] C. Shukunami , Y. Yoshimoto , A. Takimoto , H. Yamashita , Y. Hiraki , Jpn. Dent. Sci. Rev. 2016, 52, 84. 28408960 10.1016/j.jdsr.2016.04.003 PMC5390337

[6] a) C. K. Chan , E. Y. Seo , J. Y. Chen , D. Lo , A. McArdle , R. Sinha , R. Tevlin , J. Seita , J. Vincent‐Tompkins , T. Wearda , W. J. Lu , K. Senarath‐Yapa , M. T. Chung , O. Marecic , M. Tran , K. S. Yan , R. Upton , G. G. Walmsley , A. S. Lee , D. Sahoo , C. J. Kuo , I. L. Weissman , M. T. Longaker , Cell 2015, 160, 285; 25594184 10.1016/j.cell.2014.12.002 PMC4297645

[7] N. Schaum , J. Karkanias , N. F. Neff , A. P. May , S. R. Quake , T. Wyss‐Coray , S. Darmanis , J. Batson , O. Botvinnik , M. B. Chen , S. Chen , F. Green , R. C. Jones , A. Maynard , L. Penland , A. O. Pisco , R. V. Sit , G. M. Stanley , J. T. Webber , F. Zanini , A. S. Baghel , I. Bakerman , I. Bansal , D. Berdnik , B. Bilen , D. Brownfield , C. Cain , M. B. Chen , S. Chen , M. Cho , et al., Nature 2018, 562, 367.30283141

[8] a) B. Pijuan‐Sala , J. A. Griffiths , C. Guibentif , T. W. Hiscock , W. Jawaid , F. J. Calero‐Nieto , C. Mulas , X. Ibarra‐Soria , R. C. V. Tyser , D. L. L. Ho , W. Reik , S. Srinivas , B. D. Simons , J. Nichols , J. C. Marioni , B. Gottgens , Nature 2019, 566, 490; 30787436 10.1038/s41586-019-0933-9 PMC6522369

[9] a) P. J. Fabre , M. Leleu , B. Mascrez , Q. Lo Giudice , J. Cobb , D. Duboule , BMC Biol. 2018, 16, 101; 30223853 10.1186/s12915-018-0570-z PMC6142630

[10] A. Doyle , M. P. McGarry , N. A. Lee , J. J. Lee , Transgenic Res. 2012, 21, 327. 21800101 10.1007/s11248-011-9537-3 PMC3516403

[11] X. Han , H. Chen , D. Huang , H. Chen , L. Fei , C. Cheng , H. Huang , G. C. Yuan , G. Guo , Genome Biol. 2018, 19, 47. 29622030 10.1186/s13059-018-1426-0 PMC5887227

[12] D. Grun , M. J. Muraro , J. C. Boisset , K. Wiebrands , A. Lyubimova , G. Dharmadhikari , M. van den Born , J. van Es , E. Jansen , H. Clevers , E. J. P. de Koning , A. van Oudenaarden , Cell Stem Cell 2016, 19, 266. 27345837 10.1016/j.stem.2016.05.010 PMC4985539

[13] M. D. Muzumdar , B. Tasic , K. Miyamichi , L. Li , L. Luo , Genesis 2007, 45, 593. 17868096 10.1002/dvg.20335

[14] a) N. Liu , G. A. Garry , S. Li , S. Bezprozvannaya , E. Sanchez‐Ortiz , B. Chen , J. M. Shelton , P. Jaichander , R. Bassel‐Duby , E. N. Olson , Nat. Cell Biol. 2017, 19, 202; 28218909 10.1038/ncb3477 PMC5332283

[15] a) J. A. Blake , J. T. Eppig , J. A. Kadin , J. E. Richardson , C. L. Smith , C. J. Bult , Nucleic Acids Res. 2017, 45, D723; 27899570 10.1093/nar/gkw1040 PMC5210536

[16] S. Picelli , A. K. Bjorklund , O. R. Faridani , S. Sagasser , G. Winberg , R. Sandberg , Nat. Methods 2013, 10, 1096. 24056875 10.1038/nmeth.2639

[17] V. Y. Kiselev , K. Kirschner , M. T. Schaub , T. Andrews , A. Yiu , T. Chandra , K. N. Natarajan , W. Reik , M. Barahona , A. R. Green , M. Hemberg , Nat. Methods 2017, 14, 483. 28346451 10.1038/nmeth.4236 PMC5410170

[18] M. I. Love , W. Huber , S. Anders , Genome Biol. 2014, 15, 550. 25516281 10.1186/s13059-014-0550-8 PMC4302049

[19] a) T. Sueyoshi , K. Yamamoto , H. Akiyama , Matrix Biol. 2012, 31, 352; 22885149 10.1016/j.matbio.2012.07.002

[20] Z. Wang , M. Gerstein , M. Snyder , Nat. Rev. Genet. 2009, 10, 57. 19015660 10.1038/nrg2484 PMC2949280

[21] A. E. Brent , R. Schweitzer , C. J. Tabin , Cell 2003, 113, 235. 12705871 10.1016/s0092-8674(03)00268-x

[22] a) C. Shukunami , A. Takimoto , M. Oro , Y. Hiraki , Dev. Biol. 2006, 298, 234; 16876153 10.1016/j.ydbio.2006.06.036

[23] a) A. H. Huang , N. A. Motlekar , A. Stein , S. L. Diamond , E. M. Shore , R. L. Mauck , Ann. Biomed. Eng. 2008, 36, 1909; 18791827 10.1007/s10439-008-9562-4

[24] A. K. Levay , J. D. Peacock , Y. Lu , M. Koch , R. B. Hinton Jr. , K. E. Kadler , J. Lincoln , Circ. Res. 2008, 103, 948. 18802027 10.1161/CIRCRESAHA.108.177238 PMC2743952

[25] S. Agarwal , S. J. Loder , D. Cholok , J. Peterson , J. Li , C. Breuler , R. Cameron Brownley , H. Hsin Sung , M. T. Chung , N. Kamiya , S. Li , B. Zhao , V. Kaartinen , T. A. Davis , A. T. Qureshi , E. Schipani , Y. Mishina , B. Levi , Stem Cells 2017, 35, 705. 27862618 10.1002/stem.2515 PMC5529170

[26] J. A. McKenzie , E. Buettmann , A. C. Abraham , M. J. Gardner , M. J. Silva , M. L. Killian , FASEB J. 2017, 31, 882. 27864378 10.1096/fj.201600969R PMC5295726

[27] a) M. L. Killian , S. Thomopoulos , FASEB J. 2016, 30, 301; 26443819 10.1096/fj.14-258236 PMC4684511

[28] a) E. Kague , S. M. Hughes , E. A. Lawrence , S. Cross , E. Martin‐Silverstone , C. L. Hammond , Y. Hinits , FASEB J. 2019, 33, 9116. 31100023 10.1096/fj.201802654RR PMC6662971

[29] a) B. Langmead , S. L. Salzberg , Nat. Methods 2012, 9, 357; 22388286 10.1038/nmeth.1923 PMC3322381

[30] a) W. Huang da , B. T. Sherman , R. A. Lempicki , Nucleic Acids Res. 2009, 37, 1; 19033363 10.1093/nar/gkn923 PMC2615629

[31] a) E. Z. Macosko , A. Basu , R. Satija , J. Nemesh , K. Shekhar , M. Goldman , I. Tirosh , A. R. Bialas , N. Kamitaki , E. M. Martersteck , J. J. Trombetta , D. A. Weitz , J. R. Sanes , A. K. Shalek , A. Regev , S. A. McCarroll , Cell 2015, 161, 1202; 26000488 10.1016/j.cell.2015.05.002 PMC4481139

