# Supplementary material for: Atlas of Musculoskeletal Stem Cells with the Soft and Hard Tissue Differentiation Architecture
Source: Adv Sci (Weinh). 2020 Oct 22;7(23):2000938. doi: 10.1002/advs.202000938 (PMC7710003; doi:10.1002/advs.202000938)
Supplement: Supplementary file 1 — Supporting Information [file ADVS-7-2000938-s001.pdf]

© 2020 Wiley-VCH GmbH

ADVANCED SCIENCE
Open Access

Supporting Information

for *Adv. Sci.,* DOI: 10.1002/advs.202000938

Atlas of Musculoskeletal Stem Cells

with The Soft and Hard Tissue

Differentiation Architecture

Zi Yin, Junxin Lin, Ruojin Yan, Richun Liu, Mengfei Liu, Bo Zhou

, Wenyan Zhou, Chengrui An, Yangwu Chen,Yejun Hu,

Chunmei Fan, Kun Zhao, Bingbing Wu,

Xiaohui, Zou, Jin Zhang, Ahmed H. El-Hashash

, Xiao Chen, and, Hongwei Ouyang**

Supporting Information

Atlas of Musculoskeletal Stem Cells with The Soft and Hard Tissue Differentiation Architecture

Zi Yin[2,3,8#], Junxin Lin[1,3#], Ruojin Yan[1,3], Richun Liu[1,3], Mengfei Liu[1,3], Bo Zhou[1,3], Wenyan Zhou[1,3,4], Chengrui An[1,3], Yangwu Chen[1,3],Yejun Hu[1,3], Chunmei Fan[2,3]; Kun Zhao[1,3], Bingbing Wu[1,3], Xiaohui, Zou[3,6], Jin Zhang[7], Ahmed H. El-Hashash[4,9], Xiao Chen[1,3,5,8], Hongwei Ouyang[1,3,4,5,8*]*

Supplemental figures and legends

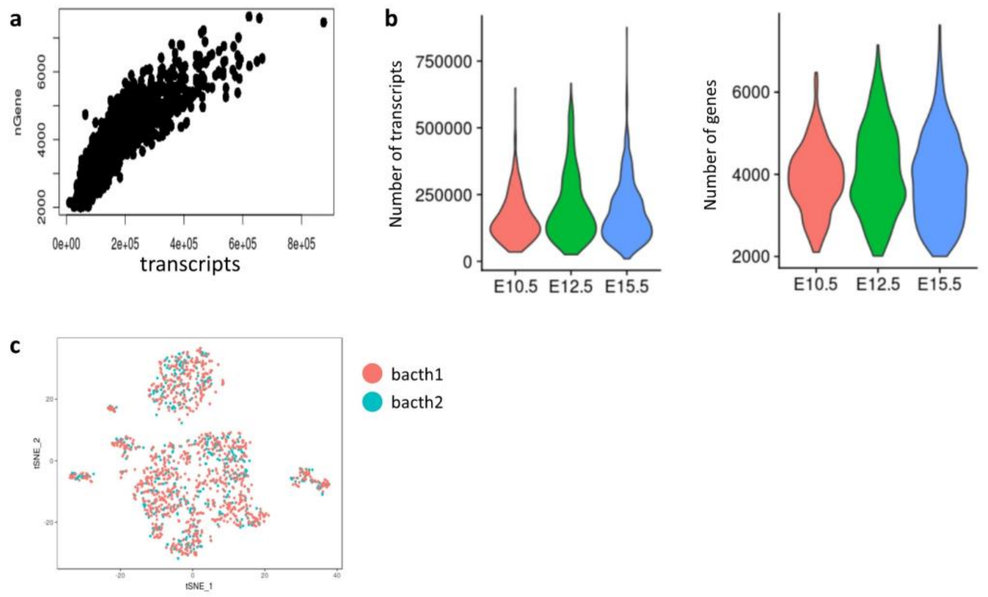

Figure S1. Quality control of the data set.

a) Scatter diagram showed the gene numbers (nGene) and transcript numbers of single cells after cell filtering.

b) Violin plots show the distribution of transcripts and genes detected per cell at E10.5, E12.5, E15.5.

c) Batch information of single cells from E10.5, E12.5, E15.5 mice hindlimb mapped on t-SNE plots.

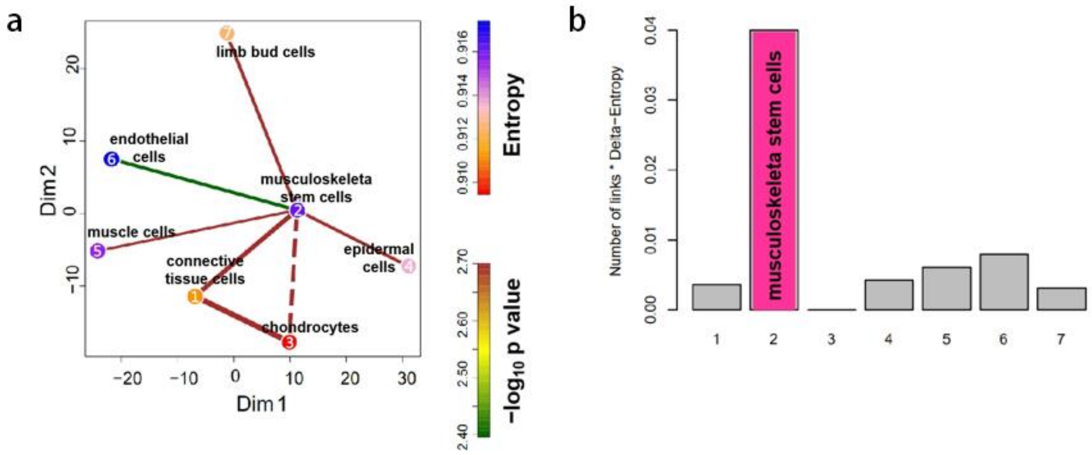

Figure S2. StemID analysis of the limb compartment exclude immune cells.

a) Inferred lineage tree. Significant links are shown ($p < 0.01$). The color of the link indicates the $-\log 10\ p$ value. The color of the vertices indicates the entropy. The thickness indicates the link score, reflecting how densely a link is covered with cells.

b) Barplot of StemID scores. The cluster number represent 1: Connective tissue cells; 2: Musculoskeletal stem cells; 3: Chondrocytes; 4: Epidermal cells; 5: Muscle cells; 6: Endothelial cells; 7: Limb bud cells.

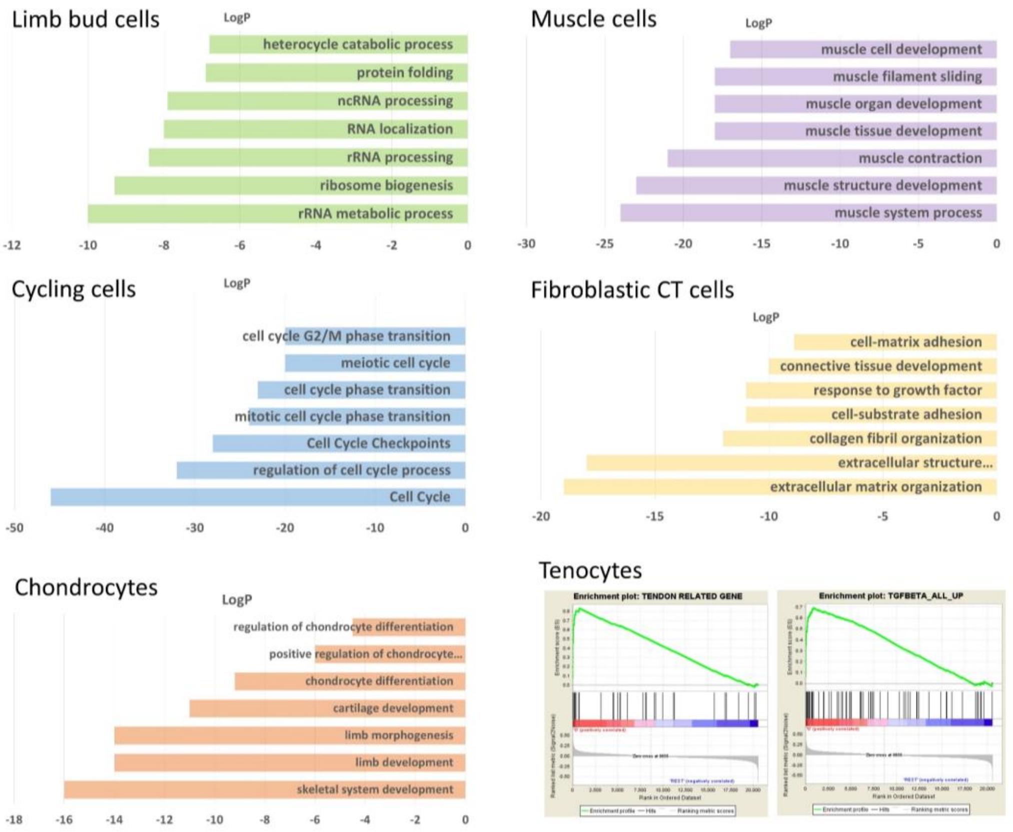

Figure S3. The enriched GO terms or GSEA results of marker genes of each *Scx* expressing cell sub-cluster.

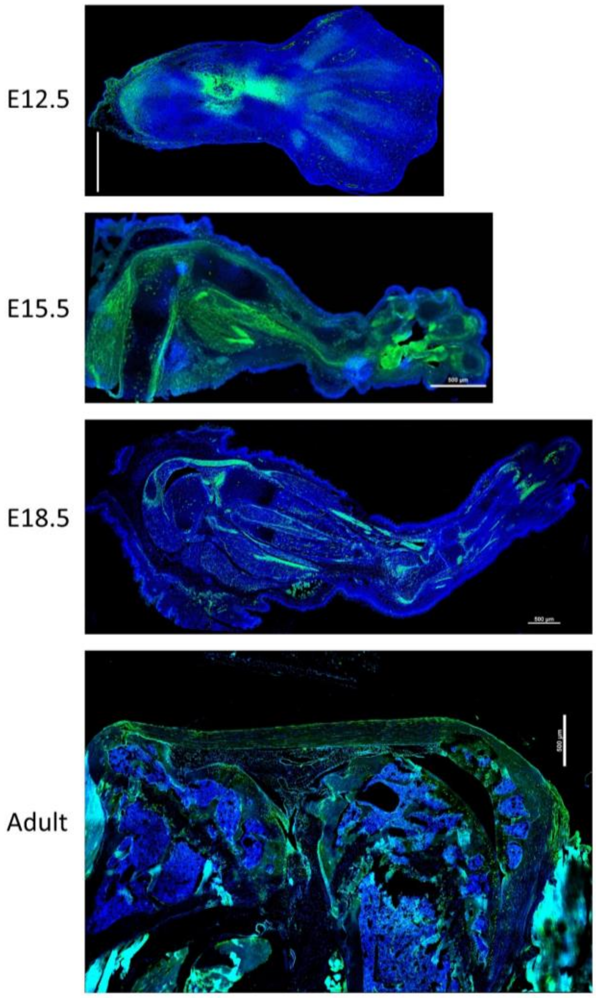

Figure S4. Immunofluorescence of Scx-GFP expression at different developmental stages. Scale bar, 500 μm.

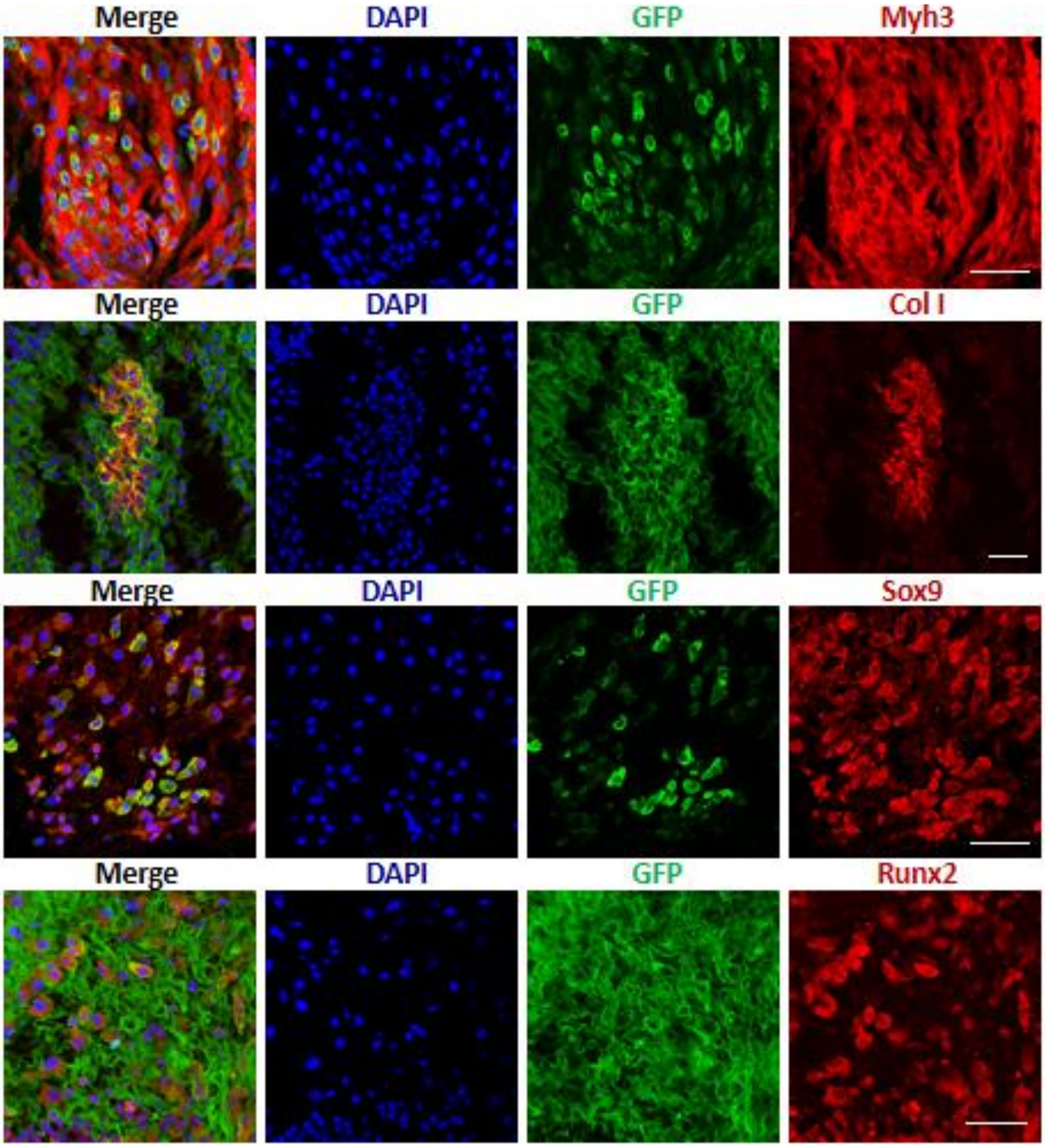

Figure S5. The immunofluorescence staining showed that injected mGFP labelling cells (representing the Scx-lineage) in developing limb bud express embryonic myofiber marker Myh3, connective tissue matrix Col1, chondrogenic marker Sox9, or osteogenic marker Runx2. Scale bar, 50 μm.

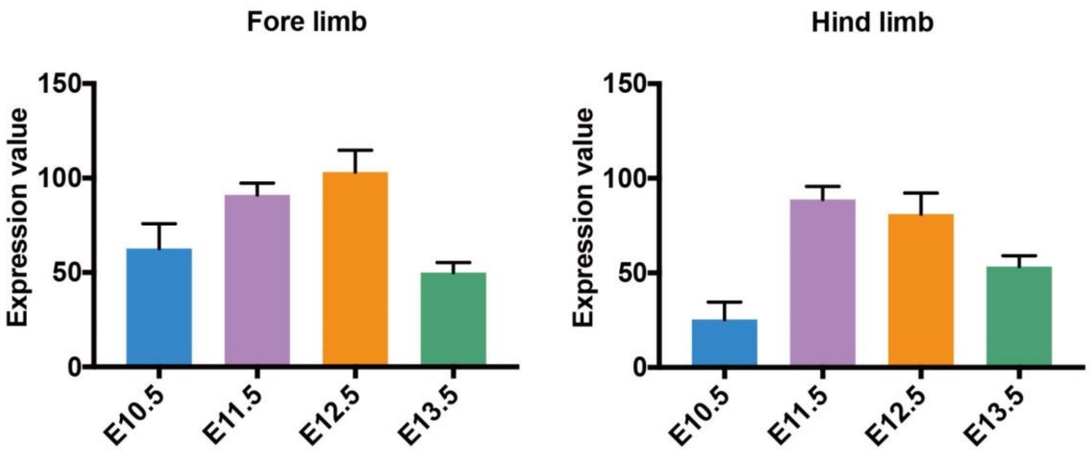

Figure S6. Gene expression of Scx during embryonic limb development Day 10.5, Day 11.5, Day 12.5 and Day 13.5.

cluster.

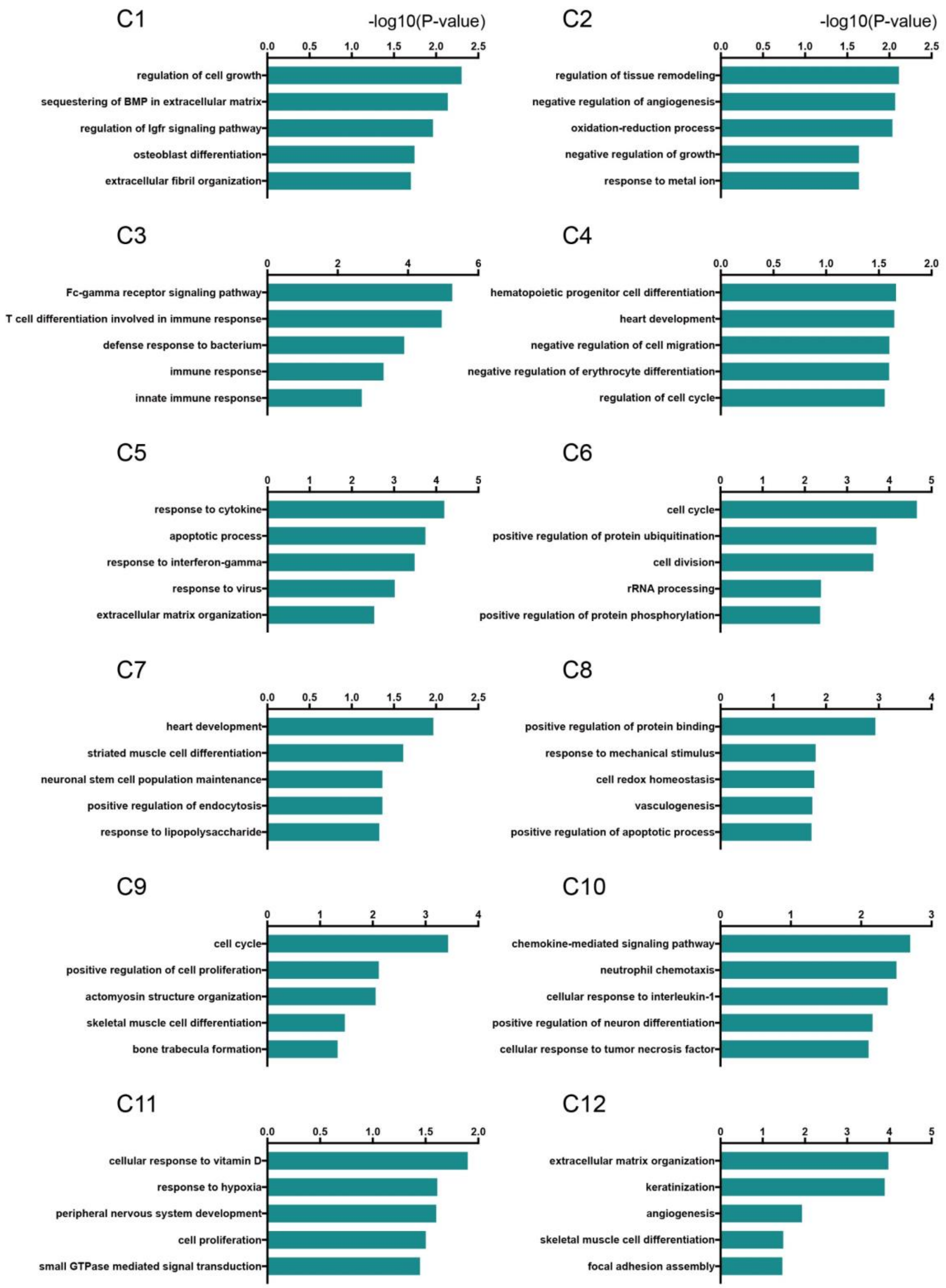

Figure S7. The enriched GO terms (biological processes) of marker genes of each cell cluster.

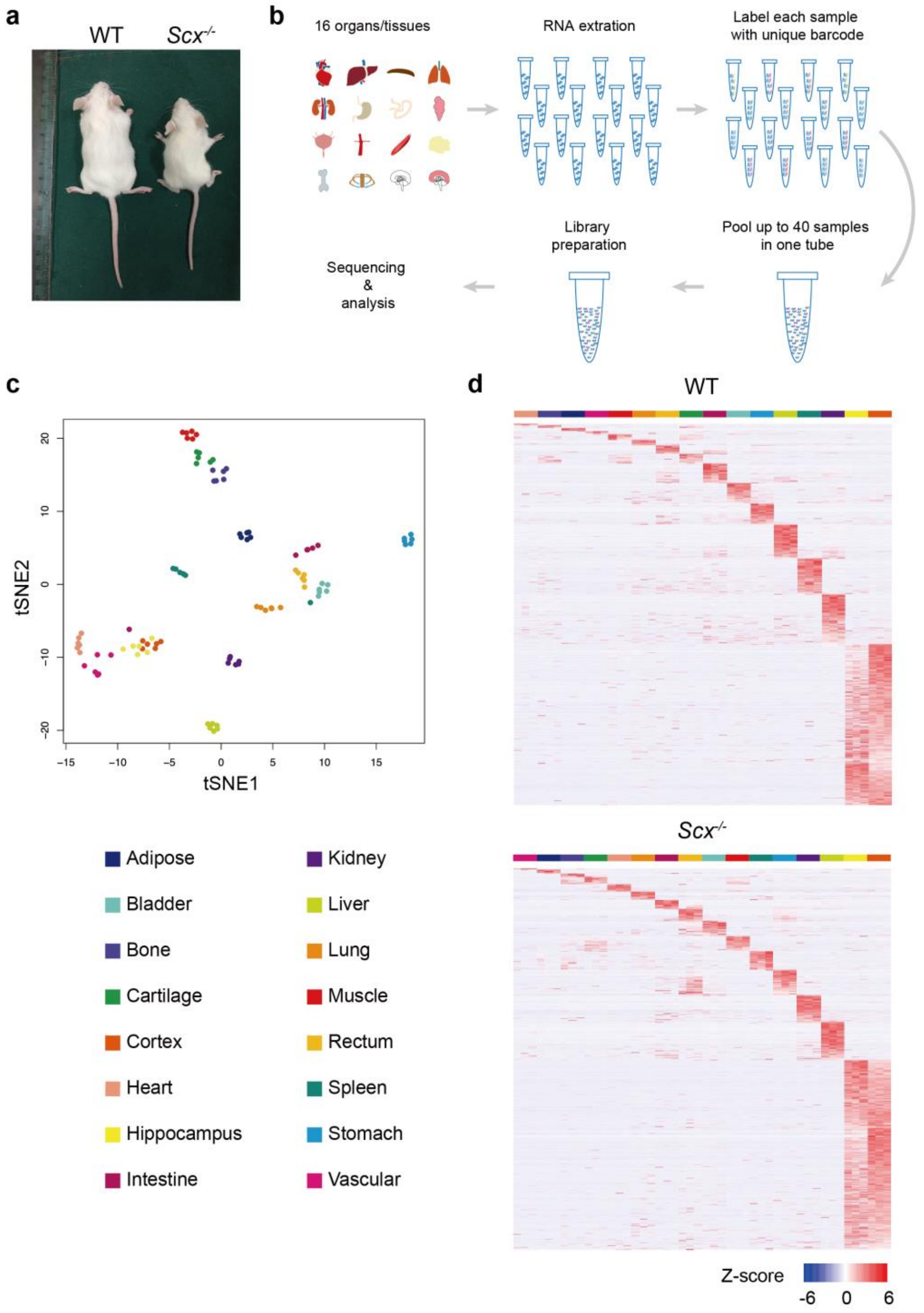

Figure S8. Multiple sample RNA-seq of 16 tissues from wild-type and *Scx*$^{-/-}$ mice.

a) The appearance of wild-type and *Scx*$^{-/-}$ mice.

b) A schematic of the basic workflow for MuSeq.

c) t-SNE maps of MuSeq data for 16 non-sexual tissue samples from wild-type and *Scx*$^{-/-}$ mice. Plots are colored by tissue type.

d) Expression profiles of tissue-enriched genes. Expression data for 3,355 (wild-type) and 3,472 (*Scx*$^{-/-}$) tissue-enriched genes were arranged by tissue type.

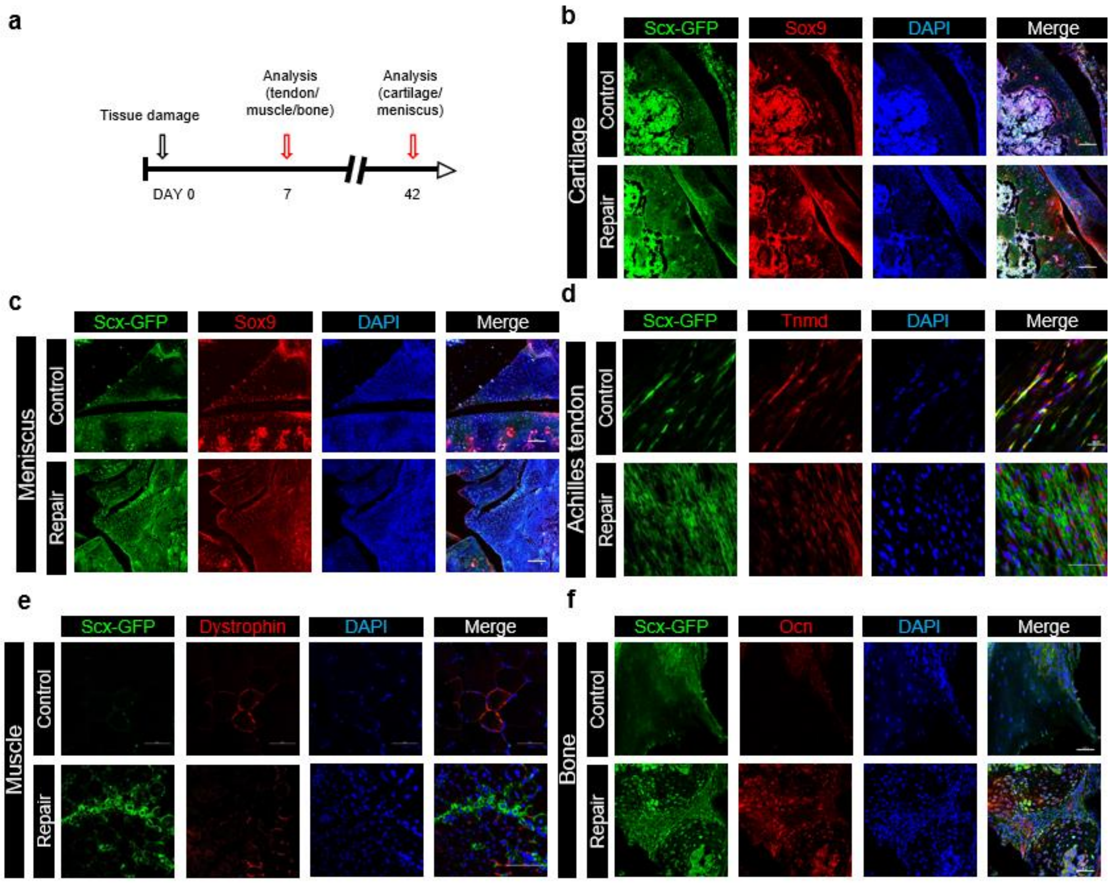

Figure S9. Contribution of Scx-positive cells during hind limb repair.

(a) Schematic illustration of articular cartilage, meniscus, Achilles tendon, tibialis anterior muscle and bone injury treatment and sample collection at designated times for adult Scx-GFP mice. Immunostaining of tissue sections showed that Scx+ cells were expressed in the articular cartilage (b), meniscus (c), Achilles tendon (d), tibialis anterior muscle (e) and bone (f) after injury and were involved in limb injury repair. Green, Scx-GFP; red, representative lineage markers; blue, nuclear staining with DAPI). Scale bar, 100 μm (articular cartilage, meniscus). Scale bar, 50 μm

(Achilles tendon, tibialis anterior muscle and bone).

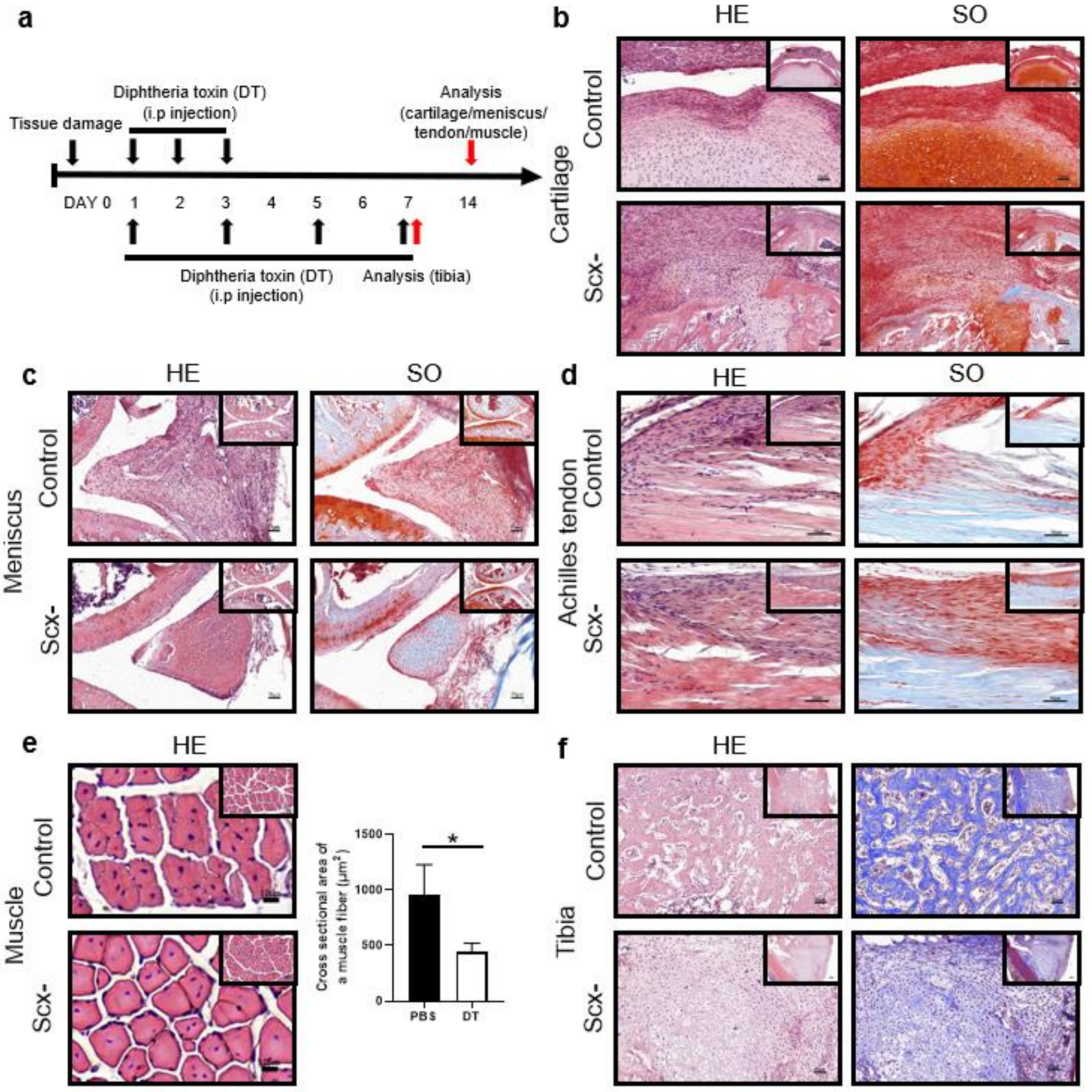

Figure S10. Scx-cell ablation impairs limb injury repair.

(a) Experimental strategy of DT administration to clear Scx+ cells in injured animal models. Histological analysis of articular cartilage injury (b), meniscus injury (c), Achilles tendon injury (d) in Scx+ cells scavenging group and control group. Scale bars, 50 μm. (e) Representative pictures of HE stained sections of tibialis anterior

muscles treated with CTX 14 days after injury and average of centrally nucleated myofiber size values in TA muscle sections. Values are mean ± SEM. Scale bars, 10 μm. (f) Analysis of HE and masson's trichrome staining in Scx+ cells scavenging group and control group in tibial injury. Scale bars, 50 μm. Data were tested for significance by an unpaired two-tailed t-test. *P < 0.05.

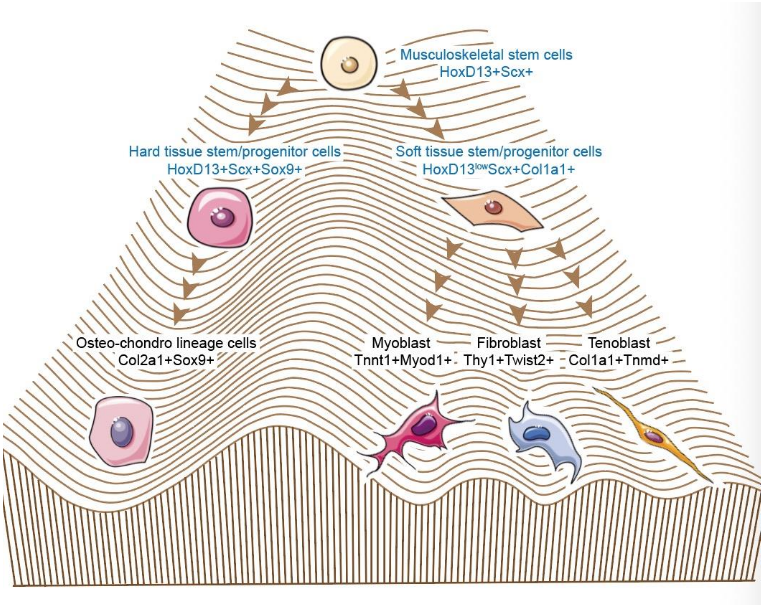

Figure S11. Musculoskeletal Stem Cells With The Soft and Hard Tissue Differentiation potential
